# An international multi-institutional validation study of the algorithm for prostate cancer detection and Gleason grading

**DOI:** 10.1038/s41698-023-00424-6

**Published:** 2023-08-15

**Authors:** Yuri Tolkach, Vlado Ovtcharov, Alexey Pryalukhin, Marie-Lisa Eich, Nadine Therese Gaisa, Martin Braun, Abdukhamid Radzhabov, Alexander Quaas, Peter Hammerer, Ansgar Dellmann, Wolfgang Hulla, Michael C. Haffner, Henning Reis, Ibrahim Fahoum, Iryna Samarska, Artem Borbat, Hoa Pham, Axel Heidenreich, Sebastian Klein, George Netto, Peter Caie, Reinhard Buettner

**Affiliations:** 1 Institute of Pathology, University Hospital Cologne, Cologne, Germany; 2 Indica Labs, Albuquerque, NM USA; 3 Institute of Pathology, Landesklinikum Wiener Neustadt, Wiener Neustadt, Austria; 4 Institute of Pathology, University Hospital Aachen, Aachen, Germany; 5 Institute of Pathology Troisdorf, Troisdorf, Germany; 6 Urology Clinic, Municipal Clinic of Brunswick, Brunswick, Germany; 7 Institute of Pathology, Municipal Clinic of Brunswick, Brunswick, Germany; 8 Divisions of Human Biology and Clinical Research, Fred Hutch Cancer Center, Seattle, WA USA; 9 Dr. Senckenberg Institute of Pathology, University Hospital Frankfurt, Goethe University Frankfurt, Frankfurt am Main, Germany; 10 Department of Pathology, Sourasky Medical Center, Tel Aviv, Israel; 11 Department of Pathology, University Hospital Maastricht, Maastricht, The Netherlands; 12 Department of Pathology, Burnasyan Federal Medical Biophysical Center of Federal Medical Biological Agency, Moscow, Russia; 13 Department of Pathology, Bach Mai Hospital, Hanoi, Vietnam; 14 Department of Pathology, University of Nagasaki, Nagasaki, Japan; 15 Clinic of Urology, University Hospital Cologne, Cologne, Germany; 16 Department of Pathology, University of Alabama, Birmingham, AL USA

**Keywords:** Pathology, Prostate cancer

## Abstract

Pathologic examination of prostate biopsies is time consuming due to the large number of slides per case. In this retrospective study, we validate a deep learning-based classifier for prostate cancer (PCA) detection and Gleason grading (AI tool) in biopsy samples. Five external cohorts of patients with multifocal prostate biopsy were analyzed from high-volume pathology institutes. A total of 5922 H&E sections representing 7473 biopsy cores from 423 patient cases (digitized using three scanners) were assessed concerning tumor detection. Two tumor-bearing datasets (core *n* = 227 and 159) were graded by an international group of pathologists including expert urologic pathologists (*n* = 11) to validate the Gleason grading classifier. The sensitivity, specificity, and NPV for the detection of tumor-bearing biopsies was in a range of 0.971–1.000, 0.875–0.976, and 0.988–1.000, respectively, across the different test cohorts. In several biopsy slides tumor tissue was correctly detected by the AI tool that was initially missed by pathologists. Most false positive misclassifications represented lesions suspicious for carcinoma or cancer mimickers. The quadratically weighted kappa levels for Gleason grading agreement for single pathologists was 0.62–0.80 (0.77 for AI tool) and 0.64–0.76 (0.72 for AI tool) for the two grading datasets, respectively. In cases where consensus for grading was reached among pathologists, kappa levels for AI tool were 0.903 and 0.855. The PCA detection classifier showed high accuracy for PCA detection in biopsy cases during external validation, independent of the institute and scanner used. High levels of agreement for Gleason grading were indistinguishable between experienced genitourinary pathologists and the AI tool.

## Introduction

Digital pathology is making its way into routine diagnostic pathology workflow. Digitization allows for more than just the signing out of cases without a microscope; and several other optimizations exist such as effective management of archived cases, easy accessibility of cases for pathological and inter-disciplinary discussions, and the automatization of many diagnostic pathology tasks.

Pathologic examination of prostate specimens is laborious and time consuming due to the large number of slides per case (50–100 slides per case). Several clinical grade, AI-based diagnostic tools and a plethora of research algorithms were recently published for tumor detection and Gleason grading of prostate cancer in histological sections^1–11^. Recently, additional applications for detection of molecular-genetic alterations based on tumor morphology have also been reported^12,13^.

Most of these studies provide appealing evidence for high diagnostic accuracy and potential integration of the tools into routine diagnostics. However, in most of these studies validation material included only cases from a small number of independent clinical centers (one or no external validation) which might question the effective generalizability of the algorithm to material from other pathology departments. Some of the other critical points during algorithm development is the necessity of large amounts of training data, quality control, and tight involvement of pathologists in all aspects of algorithm development (data curation, annotation, algorithm validation)^1,11,12,14^.

The aim of the current study is the validation of a clinical grade AI tool for prostate cancer detection and Gleason grading from prostate biopsy cases. The validation of tumor detection was carried out using large multi-institutional datasets of prostate biopsy cores from five pathology departments representing highly heterogenous pathology lab practices digitized using three different histoscanners. The validation of the Gleason grading algorithm was performed using biopsy samples from two pathology departments analyzed by 11 board-certified pathologists representing 8 different countries. We show that the performance of the AI tool for both tumor detection and Gleason grading is indistinguishable from experienced genitourinary pathologists. Our findings support that this AI tool can be effectively applied to a highly heterogeneous material from different pathology departments and digitized across different scanner types.

## Results

### The AI tool and test cohort characteristics

**Fig. 1:**
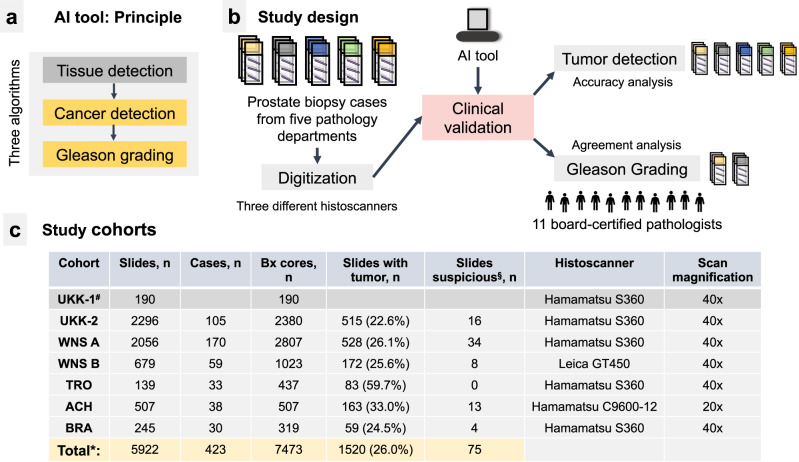
Principle of AI tool, study design, and characteristics of study cohorts. **a** The AI tool consists of tissue detection-, prostate cancer detection-, and Gleason grading-modules representing different deep learning-based algorithms. The prostate cancer detection module also detects other tissue classes, such as benign glandular, stromal tissue, high-grade prostatic intraepithelial neoplasia (HGPIN) and some others. **b** Study design includes validation of the AI tool using material from five pathology departments. Two cohorts (tumor-bearing slides) were used for validation of Gleason grading and were analyzed by 11 board-certified pathologists and AI tool. **c** Slides from five departments were included in the study. WNS B represents a subcohort of WNS A scanned by a different histoscanner. ^#^A negligibly small subset of temporally separated biopsy slides (UKK-1) was originally included into the training dataset. We provide this information for transparency. *This calculation excludes UKK-1 slides. UKK University Hospital Cologne, WNS Hospital Wiener Neustadt, TRO Institute of Pathology Troidorf, ACH University Hospital Achen, BRA Municipal Hospital Brunswick.

**Fig. 2:**
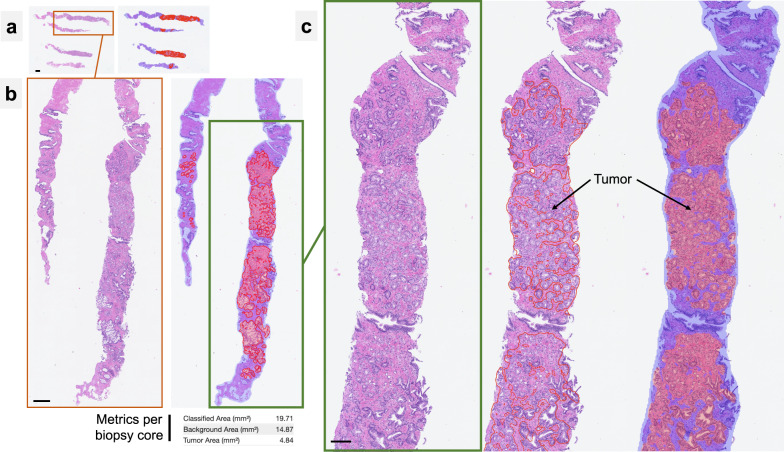
Example of outputs provided by AI tool. The example of a biopsy slide with four biopsy cores is shown under different magnifications (**a**–**c**). Tumor maps are provided as overlays or contours upon the original slides (red color: tumor, blue: detected tissue regions). The quantitative metrics (including tumor area) are generated on the per slide basis. For examples of Gleason grading algorithm output see Supplementary Figs. 1–3. Scale bars: **a** 1000 µm. **b** 200 µm. **c** 100 µm.

The AI tool for tumor detection and Gleason grading (Figs. 1a, b and 2 and Supplementary Figs. 1–3) was evaluated using six datasets from five different pathology departments (Fig. 1c). A very small subset of temporal separated biopsy cores from one large cohort (UKK-1; *n* = 190) was used to extend the training dataset; further cases from this cohort (UKK-2) with in total 2296 slides constituted a test cohort (timely separated cases, not seen by the AI tool) (Fig. 1c). The histological sections from all cohorts were digitized using Hamamatsu histoscanners (Fig. 1c) with one cohort (WNS B; subset of cases from WNS A, slides *n* = 679) digitized by both Hamamatsu and Leica scanners (Supplementary Figs. 4 and 5). With the exception of one cohort (ACH; ×20 magnification), all cohorts were digitized under ×40 objective magnification.

### Validation of tumor detection

The AI tool provides two alternative metrics for tumor detection which were used for the classification of single tumor cores as either positive for tumor or not: (1) area of region(s) detected by the algorithm as a tumor (no probability thresholding, just highest tumor probability for a region among tissue classes recognized by the algorithm) and (2) maximal probability for any of the regions within a core to be a tumor. Using a small calibration subset reserved from the training dataset we selected a threshold for both tumor area (first approach, 0.05 mm^2^) and maximal tumor tissue class probability per core (second approach, 0.85) for classifying single cores as positive for tumor. The AI tool parameters were frozen for further validation on external case cohorts.

**Fig. 3:**
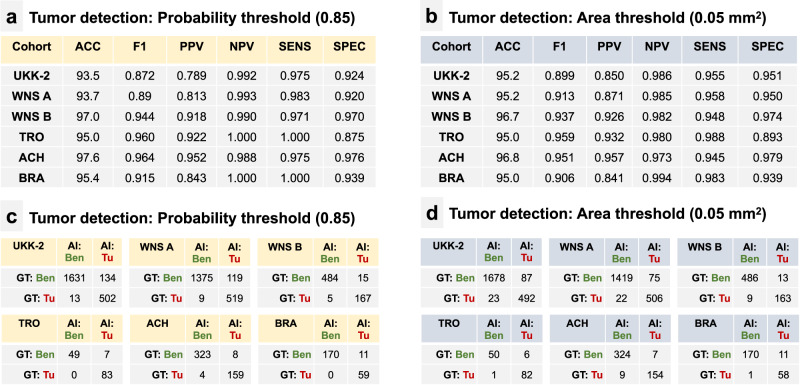
Tumor detection accuracy of AI tool. Analysis includes biopsy slides with clear classification into benign or tumor-bearing (excluding 75 slides with “suspicious” regions, s. Fig. 1c). **a** Using maximal probability of being a tumor for different regions of single slides for identification of biopsy slides with tumor tissue. These thresholds were identified on a small internal validation dataset during algorithm development. **b** Using area threshold for identification of biopsy slides with tumor tissue. Confusion matrices for single slide AI-based classification compared to ground truth information: **c** Using a probability threshold, **d** Using a tumor area threshold. ACC overall accuracy, F1 F1 score, PPV positive predictive value, NPV negative predictive value, SENS sensitivity, SPEC specificity, AI: Ben slides classified as benign by AI tool, AI: Tu slides classified as tumor-bearing by AI tool, GT: Ben ground truth: benign slides, GT: Tu ground truth: slides containing tumor.

We provide validation results separately for biopsy slides with clear classification (tumor or benign, *n* = 5847) and for slides with suspicious lesions (ASAP, *n* = 75). As for biopsy cores with a clear classification, very high levels of accuracy for tumor detection were received by both approaches for tumor presence prediction at the slide level (Fig. 3a, b). The area thresholding approach at a selected threshold (0.05 mm^2^) allowed for a sensitivity, specificity, and negative predictive value (NPV) within ranges of 0.945–0.988, 0.893–0.979, and 0.973–0.986, respectively, for the six independent test cohorts (Fig. 3a). The second approach for the detection of biopsy cores with prostate cancer (at the selected whole-slide image maximal tumor probability threshold of 85%) allowed for tumor detection with slightly higher sensitivity and negative predictive value compared to the first approach, with a sensitivity, specificity, and a NPV in a range of 0.971–1.000, 0.875–0.976, and 0.988–1.000, respectively, for the test cohorts (Fig. 3b). In two cohorts (ACH, BRA) maximal levels of sensitivity and NPV (1.0) were evident. Some variations of sensitivity and specificity are noticeable among cohorts from different institutions, in cohorts digitized by different scanner systems, and for ACH cohort that was digitized using an objective magnification of ×20. These variations are, however, from the clinical and diagnostic point of view within acceptable ranges of specificity and sensitivity (for detailed presentation of the structure of true and false positive and true and false negative slides within each test cohort, see Fig. 3c, d). On the case level, our algorithm shows sensitivity of 1.0 for tumor detection in all cohorts (Supplementary Table 6).

**Fig. 4:**
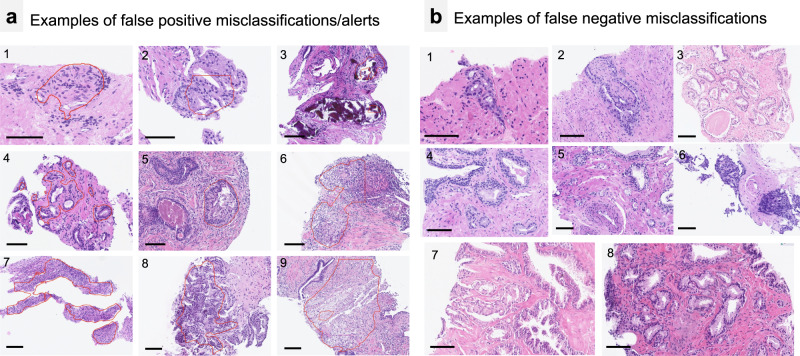
Examples of false positive and false negative misclassifications/alerts by AI tool. **a** Most false positive misclassifications/alerts are from known carcinoma mimickers: atrophic glands (4), histiocytic and inflammatory reactions (6,9), unusual luminal content (5) or complex structure of benign glands (2,8). Mechanically distorted regions (1), intraluminal calcifications (3), prominent stromal hyperplasia (7) were rarely a reason for false positive misclassifications/alerts. Such regions were interpreted as useful alerts by most pathologists. For additional examples see Supplementary Fig. 7. **b** Examples of false negative misclassifications. One of the unifying qualities of substantial number of such areas (see also Supplementary Fig. 6) were low quality of material or mechanical artifacts (3,6,7,8). Therefore, in case of artifacts or low quality of cutting and staining the predictions of the model should be interpreted with caution. Comments: All scale bars 100 µm.

Most false positive tumor misclassifications and alerts occurred in the setting of known carcinoma mimickers as well as from regions with dense histiocyte-rich inflammatory infiltrate (e.g., granulomatous prostatitis). A visual summary of these features, is shown in Fig. 4a and Supplementary Fig. 7. During review of the false positively highlighted regions, most of them were perceived by pathologists as useful alerts in regions that warrant additional attention and IHC evaluation. The analysis of biopsy slides with false negative classifications by the algorithm is summarized in Fig. 4b and Supplementary Fig. 6. This analysis did not reveal any obvious morphological similarities among misclassified tumor regions, with suboptimal tissue cutting and staining quality as well as mechanical artifacts in at least part of the cases being the only noticeable parameter. False negatively misclassified cores did not affect a case level classification in any of the cases from the cohorts. The algorithm highlighted the detection of biopsy slides containing tumor tissue that were missed by pathologists during initial evaluation. This information is available for the UKK-2 and WNS cohorts where 13 single biopsy slides containing tumor tissue, respectively, were correctly identified by the AI algorithm (representative examples in Supplementary Figs. 8 and 9).

Among the biopsy slides with unclear classification, considered as suspicious (ASAP) by pathologists during central review (*n* = 75), 10/16 (62.5%), 24/34 (70.6%), 6/8 (75.0%), 7/13 (53.9%), and 3/4 (75.0%) were nominated with a tumor alert for UKK-2, WNS-A, WNS-B, ACH, and BRA datasets respectively, showing high concordance to pathologists opinion and representing useful alerts for further clarification (e.g., with deeper levels and immunohistochemistry). Representative examples of such regions are shown in Supplementary Fig. 10.

### Gleason grading validation

Two cohorts of prostate biopsy cores containing tumor were included into the Gleason grading experiments. Two hundred forty-three whole-slide images with one or more biopsy cores containing prostate carcinoma from UKK-2 cohort (67 consecutive prostate biopsy cases) and 177 whole-slide images from WNS-A cohort (60 consecutive cases) were included in this study. The UKK-2 cohort was graded independently by a group of board-certified pathologists (*n* = 10; genitourinary pathologists *n* = 8, general surgical pathologists *n* = 2), the WNS-A cohort with the same group of pathologists plus one other board-certified genitourinary pathologist (*n* = 11). The Gleason Scoring was performed by pathologists according to the recommendations of Genitourinary Pathology Society, providing Gleason score and Gleason grade group. At that, only H&E staining was used without any knowledge of immunohistochemistry. Intraductal carcinoma was not graded when clearly identifiable. In cases of suspicion of intraductal carcinoma that would potentially change the overall Gleason score for a core or lacking confidence in carcinoma diagnosis (e.g., by very small, well differentiated, artificially changed tumor regions) the pathologists were able to exclude these cores from grading with respective comment. In cases containing several biopsy cores in a single slide, a global grading over all cores within the slide was provided by graders. In total, 227 and 159 slides with one or more biopsy cores were graded by all pathologists from UKK and WNS cohorts, respectively.

**Fig. 5:**
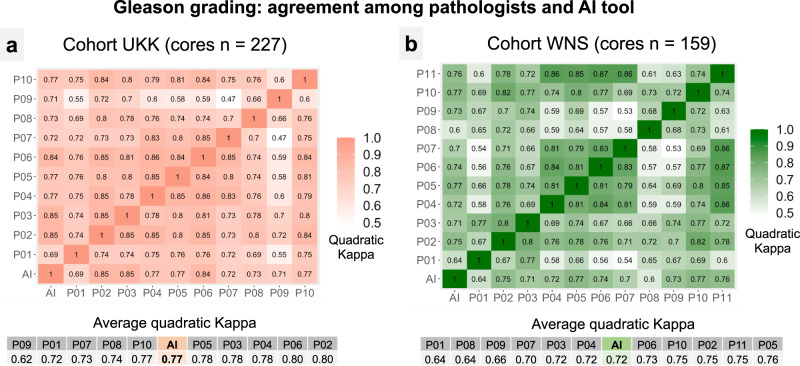
Gleason grading: agreement analysis between pathologists and AI tool. Two datasets of tumor-containing biopsy slides were used for this analysis (consecutive cases from UKK-2 and WNS A cohorts). Quadratically weighted kappa statistics was used for calculation of agreement. Presented are comparisons between single graders (UKK: 10 board-certified pathologists and AI tool; WNS: 11 board-certified pathologists and AI tool) as well as average quadratically weighted kappa levels for single graders. Pathologists 1 and 9 (P01 and P09) are general surgical pathologists working routinely with prostate cases. All other pathologists are experienced genitourinary pathologists. AI Tool performs on par with pathologists. For distribution of Gleason grade groups in cohorts see Fig. 6a. **a** Cohort UKK. **b** Cohort WNS.

**Fig. 6:**
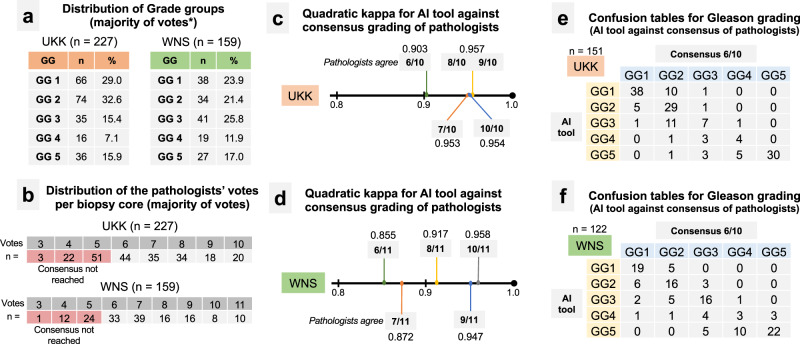
Gleason grading: distribution of Gleason grade groups among cohorts and performance of AI tool versus consensus grading of pathologists. **a** Distribution of Gleason grade groups in UKK and WNS cohorts of tumor-containing biopsy slides. This is generated using majority of votes of graders (see also **b** for distribution of votes of pathologists). As in UKK cohort (graded by ten pathologists) ~30 cases have equivocal quantity of majority votes for two grade groups simultaneously, grading of AI tool was added to produce unambiguous distribution data. For WNS cohort (graded by 11 pathologists) majority of votes classification resulted in unambiguous classification without adding AI tool grading results. Both cohorts were representative for all Gleason grade groups. **b** Distribution of the pathologists’ votes for single grade group per biopsy core (excluding AI tool). Consensus was considered in cases where at least 6 pathology votes were for a single grade group. **c**, **d** Quadratically weighted kappa levels for AI tool-provided grading results vs. grading results of pathologists in cases where consensus was reached (at least 6 votes for single grade groups), **c** UKK cohort, **d** WNS cohort. Substantial increases of agreement are evident for AI tool in cases where higher levels of consensus among pathologists are present. Confusion tables for UKK (**e**) and WNS (**f**) cohorts for cases where at least 6 pathologists were agreeing on the Gleason grading of single cores (consensus cases). GG grade group.

First, we systematically compared the grading results between single graders (pathologists and AI tool). The quadratically weighted kappa levels for single pathologists ranged 0.62–0.80 (0.77 for AI tool) and 0.64–0.76 (0.72 for AI tool) for UKK and WNS cohorts, correspondingly (Fig. 5a, b). Some differences in composition of Gleason grade groups (Fig. 6a) might be responsible for slight global differences in agreement levels among graders on these two cohorts. In general, there was a trend to higher graded cases in WNS cohort (Fig. 6a). Moreover, we investigated the influence of absolute area occupied by tumor tissue (AI tool-based estimates) on differences in grading agreement between two cohorts (Supplementary Fig. 11). A positive correlation (Pearson’s *p* 0.14, *p* = 0.03 and *p* 0.24, *p* = 0.003) between absolute tumor area in a slides and higher agreement between single graders was evident, however, this was rather weak in effect magnitude. There was also no effect of the number of biopsy cores in single slides (UKK: single core slides *n* = 198, multiple cores *n* = 29; WNS: *n* = 120, *n* = 39) on the concordance levels among pathologists (Wilcoxon test *p* > 0.5 for both cohorts).

**Fig. 7:**
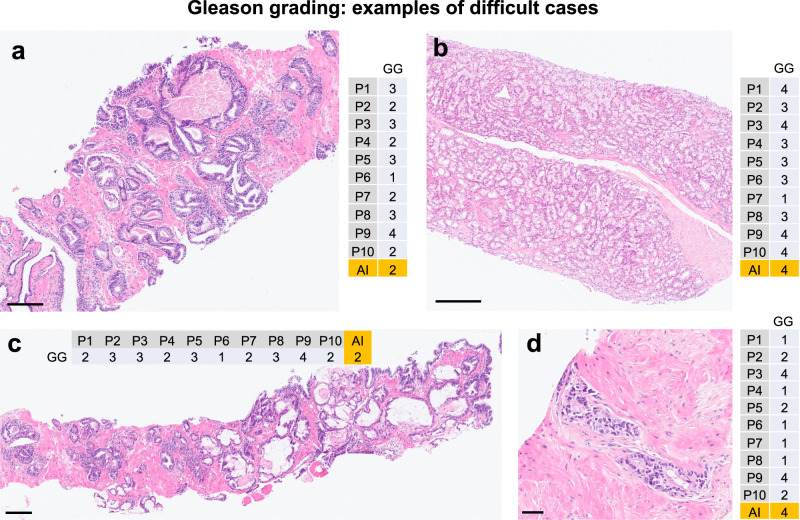
Examples of cases with discrepant Gleason grading where consensus among pathologists was not reached. **a** Case 1. **b** Case 2. **c** Case 3. **d** Case 4. Comments: Consensus is defined as at least six pathologists providing the same grade group; scale bars: **a** 100 µm. **b** 200 µm. **c** 200 µm. **d** 50 µm.

Next, we analyzed subsets of 151 and 122 whole-slide images from UKK and WNS cohorts, respectively, where consensus concerning grading was reached with at least six pathologists providing the same grade group (Fig. 6c, d). Quadratically weighted kappa levels for AI tool vs. consensus opinion of pathologists were substantially higher in such cases (UKK: 0.903, WNS: 0.855) with even higher agreement (up to 0.958) when more than 6 pathologists agreed on Gleason score of single slides (Fig. 6c). Confusion tables for grading results are provided in Fig. 6e, f and Supplementary Fig. 12A, B. Agreement levels, when biopsies were stratified according to GG1 vs. GG2-5, was also high, especially in cases where consensus could be reached among pathologists (Supplementary Fig. 12C, D). Detailed information on grading results of biopsies where consensus among pathologists was not reached is presented in Supplementary Figs. 13 and 14. Additionally, we performed an analysis of how grading of single pathologists and AI tool compares, when these single pathologists are not included into consensus grading calculation (Supplementary Fig. 15). Representative cases with major disagreement among graders are provided in Fig. 7, with disagreement mostly stemming from well-known, subjective interpretation of gland architecture.

### Comparison to publicly available algorithms

Additionally, we tested a state-of-the-art publicly available algorithm (winning algorithm from recent, large PANDA challenge to prostate cancer detection and Gleason grading^8^) on our tumor detection and Gleason grading datasets (details to implementation in Supplementary Methods). The performance of PANDA algorithm for Gleason grading was inferior compared to our algorithm (for PANDA and developed algorithm, respectively, quadratically weighted kappa for WNS dataset: 0.69 vs. 0.72, for UKK dataset 0.72 vs. 0.77; Supplementary Table 4) with substantially inferior results for tumor detection (Supplementary Table 3).

## Discussion

Prostate cancer detection in histological sections of multiregional prostate biopsies and Gleason grading of the detected carcinoma are routine, laborious pathology tasks. Artificial intelligence-based algorithms proved to be accurate tools in many tumor types, including prostate cancer^1–11,15^. In this study, we clinically validate an AI-based tool (recently received an CE-IVD certification) for tumor detection and Gleason grading in histological sections of prostate core biopsies (Figs. 1a, b and 2 and Supplementary Figs. 1–3). This validation study is one of the largest clinical validation studies of AI tool for digital pathology to date. It includes 6 datasets stemming from 5 pathology departments and comprising >5900 diagnostic slides, scanned with three different scanners and at two different magnifications (Fig. 1c). The amount of heterogeneity concerning different lab techniques, quality of cutting, staining, digitization, captured by the study cohort is enormous (Supplementary Figs. 4 and 5) and represents “real-world” practice without pre-selection of cases.

The AI tool showed a high accuracy for prostate adenocarcinoma detection. In the study we tested two slightly different approaches to render single biopsy cores positive or negative for tumor (Fig. 3). Both approaches provided very similar tumor detection accuracy metrics (Fig. 3a, b). However, our approach, using aggregated maximal probability of tissue regions being a tumor systematically provided better balance between very high sensitivity (0.975–1.000) and negative predictive value (0.988–1.000) and high specificity. This was true in all six independent datasets used for validation. High NPVs/sensitivity are naturally of particular importance for routine diagnostic cases. Importantly, an additional value of the AI tool was demonstrated by its detection of biopsy cores containing tumor tissue that was missed by pathologists during initial review (up to 13 cores per cohort, see “Results”). Even if it did not have any implications for the whole case status in our study, it might certainly have, especially in pathology departments not sub-specialized in genitourinary pathology.

Most false positive tumor misclassifications issued by the AI tool stemmed from known mimickers of carcinoma or morphologically complex regions representing useful alerts for pathologists in clinical practice (Fig. 4a and Supplementary Fig. 7). False negative tumor detections were occasionally evident with at least some of them arising in regions with mechanical/cutting artifacts or other quality control issues (out-of-focus regions), a known problem for AI-based algorithms^14^. This warrants two strategies to be implemented. First, AI tool predictions in context of any artifacts should be interpreted by pathologists with additional caution. Second, using an automatized quality control tool before processing slides with tumor detection algorithm might be of additional benefit as the former will identify and highlight or mask all artificially changed regions during the tumor detection step.

Several studies published to date have validated clinical grade AI-based algorithms for prostate cancer detection in histological sections using external data, summarized in Supplementary Table 1. Campanella et al.^1^ developed an algorithm based on the weakly supervised approach using 12,132 core needle biopsy slides which was validated using external dataset of another 12,727 slides reaching AUROC of 0.986. The AUROC might be a suboptimal metric for diagnostic tools in certain circumstances^16^ and does not allow a direct comparison to the results of the actual study as we use a fixed threshold (AUROC value for our tool in the development study was 0.992^11^). An updated version of the Campanella et al. algorithm was validated clinically in three studies^2,10,17^. In the study of Raciti et al.^17^ a dataset consisting of 232 slides (slide with tumor *n* = 93, without intraductal carcinoma). The sensitivity and specificity of the algorithm for detection of “suspicious” slides was 0.96 (4/93 slides with tumor missed) and 0.98, respectively. Authors show improvements of pathologists’ sensitivity using the same cases after a wash-out period of 4 weeks when assisted by algorithm. In the study of da Silva et al.^2^, the sensitivity and specificity on a dataset containing 579 slides from 100 patients was 0.99 and 0.93, respectively, with some slides excluded from analysis due to disagreement of pathologists on the ground truth status. In the study of Perincheri et al.^10^ algorithm reached sensitivity of 0.977 and specificity of 0.993 for detection of “suspicious” biopsy slides (*n* = 1876). In all three studies all slides originated from one pathology department, respectively. Importantly, the algorithm used in these three studies^2,10,17^ does not detect tumor, but renders slide as suspicious (presence of any of the following lesions: tumor, focal glandular atypia, atypical small acinar proliferation, high-grade prostatic intraepithelial neoplasia with adjacent atypical glands—conditions with high interobserver variability and interpretability) which prevents exact comparison to our results (we concentrated on only tumor detection). Even so, the AI tool in our study (>5900 diagnostic slides, >420 patient cases from five pathology departments) show similar performance with high, diagnostically meaningful accuracy metrics for tumor detection. Also, in the slides with unclear classification which were considered as suspicious by pathologists, the AI tool provides positive alerts in a substantial number of cases (53.9%–75.0% dependent on test cohort) allowing for high awareness levels to such regions among pathologists.

One other clinical grade algorithm was validated in a study by Pantanowitz et al.^9^. The sensitivity and specificity on the internal (same institute as training data, slides *n* = 2501) and external (slides *n* = 355) datasets was 0.996 and 0.901 and 0.985 and 0.973, respectively. Importantly, authors used additional slides from external dataset to first calibrate the algorithm to this external dataset (to digitization, staining parameters, tissue quality, etc.) which is not a typical practice. Therefore, the real generalization capabilities of the algorithm to new/external data could not be estimated based on this study. The parameters of our algorithm were frozen at the beginning of the study without any forms of accommodation of the algorithm to external data, which is also a regulatory requirement for clinical-grade tools.

In one other study by Ström et al.^7^, authors report sensitivity of 99.6% and specificity of 86.6% on the reserved internal validation dataset (enriched for high-grade cases). Both studies used original semiautomatic labeling techniques for annotation creation.

The second diagnostic aspect of our study is AI-based Gleason grading. Using two external sets of biopsy cores (slides *n* = 227 and 159) representative of all Gleason grade groups and a large, international group of board-certified pathologists (*n* = 11; 2 general surgical pathologists, 9 experiences genitourinary pathologists) representing diagnostic practices of different countries (Germany, Austria, USA, Netherlands, Israel, Japan, Vietnam, Russia) we showed that the developed algorithm performs on par with experienced genitourinary pathologists (Figs. 5 and 6). The average quadratically weighted kappa value for the AI tool was 0.77 in the first cohort (UKK; pathologists average kappa values 0.62–0.80) and 0.72 in the second cohort (WNS; pathologists average kappa values 0.64–0.76). Moreover, the agreement between the AI tool and pathologists was especially high in cases where consensus among pathologists could be reached (>0.855; Fig. 6c, d). Also, for the diagnostically critical Gleason grade group 1 (Gleason Score 3 + 3 = 6; clinical decision: active surveillance vs. active therapy) the AI tool showed similarly high levels of agreement to participating pathologists (Supplementary Fig. 12C, D). Several large studies evaluated performance of AI-based tools for prostate cancer Gleason grading against human pathologists in a controlled setting using external datasets^5,7^, summarized in Supplementary Table 2. Studies by Strom et al.^7^ and Bulten et al.^5^ show similar levels of agreement compared to our study among pathologists and the AI tool in external validation datasets (kappa levels 0.60–0.72). Some other studies showed that pathologists assisted by AI algorithms can provide more concordant and reliable grading^6,15^, mirroring the real diagnostic benefits of complementary human-AI tool interaction within a diagnostic process. Moreover, one large computational challenge (PANDA) addressed the development of Gleason grading algorithms in a competitive manner releasing large datasets for training and validation^8^. In our study, we compared the developed algorithm with a winning solution of PANDA challenge (Supplementary Tables 3 and 4) showing superiority of our algorithm. To facilitate further academic research in the area of Gleason grading and interoperability studies of algorithms, we release part of our Gleason Grading datasets (WNS, UKK) with accompanying grading results by pathologists.

Our study is not devoid of limitations. All cohorts analyzed in the study are retrospectively gathered archived cases. Further prospective evaluation with integration of the AI tool into diagnostic routine of pathologists is necessary. The optimal ways of interaction between human pathologists and AI tools to achieve maximal complementary effects is still an open field of research. Issues such as a overly high confidence of pathologists in the predictions of AI tool should be addressed by prospective evaluation. Although this study is one of the largest validation studies of AI tools for digital pathology to date including 5 different departments, the heterogeneity of pathology practice is huge in the real world. Additional validation with inclusion of more pathology departments is warranted. The AI tool might still make diagnostic mistakes and misses tumor, as human diagnosing also does. Further (continuous) development with the inclusion of difficult cases into the training data is a typical way to mitigate this problem. We used a small part of cases from one department to extend our training data. These cases were temporally separated from the cases included into the test dataset and represent a negligibly small volume of training material compared to the training dataset and to the size of the remaining test dataset. We did not see any effects on the accuracy of the algorithm on the compromised dataset, especially compared to four other, completely independent, external test datasets.

In this large, multi-institutional, international study we validate a clinical grade AI tool for prostate cancer detection and Gleason grading on biopsy material from 5 pathology departments, digitized with three different scanners at two different magnifications. We show high levels of diagnostic accuracy for prostate cancer detection and agreement levels for Gleason grading comparable with experienced genitourinary pathologists.

## Methods

### Patient cohorts, materials

Five independent cohorts of archived pathological patient cases from large academic pathology departments with sub-specialization in genitourinary pathology were used for this study: UKK—University Hospital Cologne, Germany, WNS—Hospital Wiener Neustadt, Austria, TRO—Pathology institute Troisdorf, Germany, ACH—University Hospital Aachen, Germany, BRA—Municipal Hospital Brunswick, Germany (Fig. 1c). All cases were primary multifocal prostate biopsies from patients with a suspicion of prostate cancer without prior therapy. From one large cohort (University Hospital Cologne, UKK, *n* = 2486 cores) a very small subset of slides (UKK-1, *n* = 190 cores, all cases temporally separated with at least 9 months from cases used for further test) were included in the training dataset. Where possible, consecutive biopsy cases were used without pre-selection.

### Establishing ground truth

All biopsy cores from all cohorts were centrally reviewed by experienced uropathologists for the presence of tumor (YT, AP, AQ). Corresponding immunohistochemistry (IHC) results were also reviewed when available. This multi-pathologist review of the retrospective diagnostic material was used to establish the ground truth (tumor or benign). High-grade prostatic intraepithelial neoplasia (HGPIN) lesions were classified as benign, although the algorithm recognizes HGPIN as a separate tissue class. During central review, some biopsy cores were classified as “suspicious” of carcinoma due to the presence of atypical small acinar proliferation (ASAP). In most situations, the suspicious region was either no longer present in corresponding IHC stains, the IHC slides were not available, or IHC results were inconclusive prohibiting final classification as tumor or benign. These biopsy slides (*n* = 75, Fig. 1c) were addressed in a separate sub-analysis.

### Histological slide digitization

Three case cohorts (UKK, TRO, BRA) were digitized using a NanoZoomer S360 histoscanner (Hamamatsu, Japan; resolution micron per pixel (mpp) = 0.2305), one cohort (ACH) was digitized using a NanoZoomer C9600-12 (mpp = 0.4516; this scanner allows only for scanning at magnification ×200), and one other cohort (WNS) was digitized using both a NanoZoomer S360 (mpp = 0.2305) and a Leica Aperio GT450 (Leica Biosystems, Wetzlar, Germany; mpp = 0.26). All histoscanners were maintained according to the manufacturers´ instructions and underwent regular technical servicing. All pen marks were removed from the specimens before digitization. A small number of slides were excluded from the study due to digitization issues (broken glass, out-of-focus regions, failed digitization, and histoscanners not accepting slides due to unknown reasons).

### Description of AI tool

The AI tool for prostate cancer detection and grading was developed by Indica Labs (HALO Prostate AI®, Albuquerque, NM, USA) and is a CE-IVD certified assistive tool for pathological diagnosis (Fig. 2). Briefly, the tool consists of three AI modules: tissue detection, prostate cancer detection, and Gleason grading algorithms. The two first algorithms (tissue detection, and tumor detection) are based on deep learning principle and semantic segmentation convolutional neural networks. The Gleason grading module represents a classification convolutional neural network. The tumor detection and Gleason grading are carried out at a resolution 1.5 µm/px, roughly corresponding to ×50 optical magnification. The AI tool was developed using more than 800,000 single image tiles generated from manually annotated whole-slide images. These included a large radical prostatectomy dataset^11^ as well as additional whole-slide images from the Institute of Pathology of the University Hospital of Cologne containing seminal vesicle tissue (*n* = 50; radical prostatectomy specimens) and a small set of whole-slide images of prostate biopsy cores with and without tumor (*n* = 190, UKK-1 dataset, Fig. 1c) to enrich the training dataset for biopsy tissue containing some specific artifacts. The AI tool does not utilize any stain normalization or style transfer approaches for compensation of inter-institutional variability. The AI tool is implemented within the HALO AP IMS and case viewer system (Indica Labs, CE-IVD certification for digital diagnosis in pathology).

### Principles of validation, statistical analysis

The AI tool provides several types of output: (1) color maps (red color) for regions with a high probability of being prostate cancer (overlaid on the original whole-slide images); (2) color-coded maps representing Gleason grading estimates (yellow—Gleason Pattern (GP) 3, orange—GP4, red—GP5), and (3) a number of computed metrics, whole-slide level: tissue area, tumor area, maximal tumor probability, Gleason pattern area (GP3, GP4, GP5), Gleason Score, and Gleason Grade Group. The AI tool’s performance for tumor detection was assessed at the slide level using typical accuracy metrics for computer vision studies (overall accuracy, F1 score, sensitivity, specificity, positive and negative prediction values). Gleason grading accuracy/agreement analysis was compared between participating pathologists and the AI tool at the slide level (Gleason Grade Groups), and also using consensus Gleason scores derived for a group of pathologists. Agreement analysis was performed using quadratically weighted kappa statistics in irr package for R, considering the distance between grade group classifications in cases of disagreement (and outlining biological and prognostic relevance of the discrepancy). All the statistical analyses were made in R version 4.0.3 (The R Foundation for Statistical Computing). This study was performed in adherence to STARD guidelines (“Standards for Reporting Diagnostic Accuracy studies”; s. Checklist in Supplementary Data).

### Using PANDA challenge data for validation purposes

To replicate the PANDA algorithm, we based our code on their public code (see “Data availability” section) We made several modifications to get better performance on our validation sets. First, we had the classifier run on all the tiles that passed a tissue threshold as opposed to picking the darkest 36/64 tiles for their ensembles model1/model2 respectively. Second instead of using the second pyramidal level for analysis we used a fixed resolution of 1.9 um/px. This was the approximate resolution the classifier was trained on. Since some of the validation set was scanned at a higher resolution then the pandas set, using the second level would have resulted in images of ~1.0 um/px. Lastly, we only used the ensemble corresponding to model 2 for the Gleason grading as using both led to inferior results. Using both did improve tumor detection scores, however, and we report the tumor detection for the full ensemble.

### Ethical approval

All study steps were performed in accordance with the Declaration of Helsinki. This study was approved by the Ethical Committee of the University of Cologne (20-1583), Ethical Committee of Lower Austria (GS1-EK-4/694-2021), Ethical Committee of the University of Aachen (EK 405/21), Ethical Committee of the Medical Council of Lower Saxony region, Germany (30/51/2021), and Ethical Committee of the Medical Council of the Nordrhein region, Germany (355/2021). Necessity for obtaining patients’ informed consent has been waived due to the fully retrospective and archived nature of materials used in this study.

### Reporting summary

Further information on research design is available in the Nature Research Reporting Summary linked to this article.

### Supplementary information

Supplementary Material

Reporting Summary

## Data Availability

A part of each Gleason Grading datasets (UKK and WNS; 50 biopsies/dataset, anonymized, balanced for Gleason grade groups) with grading results by 10(11) pathologists was publicly released for academic research only at Zenodo (UKK cohort: https://zenodo.org/record/8102833; WNS: https://zenodo.org/record/8102929). The images are in the OME-TIFF format at original resolution (×40) and publicly available for download. The sheet with grading results can be received from the corresponding author (Y.T.) after completing a request form (available in Zenodo-repositorium). The data (images and grading results) can be used for academic research purposes only, no commercial use is allowed. The full datasets for tumor detection and Gleason grading can be requested from corresponding author (Y.T.).

## Code availability

There is no code to be shared withing this study as the algorithm used represents a proprietary tool. The code for implementation of publicly available PANDA algorithm that was used in this study is available at https://github.com/kentaroy47/Kaggle-PANDA-1st-place-solution.
