# Supplementary material for: An international multi-institutional validation study of the algorithm for prostate cancer detection and Gleason grading
Source: NPJ Precis Oncol. 2023 Aug 15;7:77. doi: 10.1038/s41698-023-00424-6 (PMC10427608; doi:10.1038/s41698-023-00424-6)
Supplement: Supplementary file 1 — Supplementary Material [file 41698_2023_424_MOESM1_ESM.pdf]

Supplementary Table 1 Summary of the results from published studies to prostate cancer detection (clinical grade algorithms)

	Departments n	Slides n	Patients n	Algorithm sensitivity	Algorithm specificity	Comments	Ref.
Raciti et al. 2020	1 (external)	232	93	0.96	0.98	IDCP cases excluded	[18]
Da Silva et al. 2021	1 (external)	579	100	0.99	0.93	Some slides excluded if no consensus on diagnosis	[17]
Perincheri et al. 2021	1 (external)	1876	118	0.977	0.993	Detection of „suspicious“ (tumor, FGA, ASAP, PINATYP) slides, not tumor slides	[12]
Pantanowitz et al. 2020	1 internal set (same as training data)	2501	210	0.996	0.901		[11]
	1 External set (after algorithm calibration to this dataset)	355	100	0.985	0.973	Calibration	[11]

Supplementary Table 2 Summary of the results from published studies to Gleason grading (using external validation cohort)

	Departments n	Slides n	Patients n	Pathologists n	Kappa AI	Kappa pathologists	Comments	Ref.
Ström et al. 2020	1 (internal test set)	1631	246	1	0.83	-		[9]
	1 (external test set)	330	73	1	0.70	-		[9]
	1 (external test set)	87		23	0.62	0.60-0.72	ImageBase ISUP	[9]
Bulten et al. 2020	1 (internal test set)	100		15				[7]
	1 (external test set)	245		2	Vs. P1: 0.707 Vs. P2: 0.723	P1 vs P2: 0.71	Tissue microarrays	[7]

Supplementary Table 3
Direct comparison of winning PANDA challenge algorithm and developed algorithm
Task: tumor detection.

**Tumor detection dataset**	**PANDA algorithm* [ACC, F1, PPV, NPV, SENS, SPEC]**	**OWN algorithm [ACC, F1, PPV, NPV, SENS, SPEC]**
**ACH**	[0.917, 0.907, 0.918, 0.949, 0.896, 0.961]	[0.935, 0.872, 0.789, 0.992, 0.975, 0.924]
**BRA**	[0.742, 0.656, 0.488, 1.000, 1.000, 0.657]	[0.937, 0.890, 0.813, 0.993, 0.983, 0.920]
**TRO**	[0.943, 0.951, 0.975, 0.900, 0.928, 0.964]	[0.970, 0.944, 0.918, 0.990, 0.971, 0.970]
**UKK**	[0.821, 0.710, 0.560, 0.988, 0.969, 0.778]	[0.950, 0.960, 0.922, 1.000, 1.000, 0.875]
**WNS HAMA**	[0.876, 0.791, 0.708, 0.959, 0.896, 0.869]	[0.976, 0.964, 0.952, 0.988, 0.975, 0.976]
**WNS LEICA**	[0.753, 0.675, 0.509, 1.000, 1.000, 0.667]	[0.954, 0.915, 0.843, 1.000, 1.000, 0.939]

Comment: *All data presented for PANDA winning algorithm is for default cutoff 0.5 (as used in competition). Using cut-off of 1.0 led to substantially worse results (regarding sensitivity and PPV).

Supplementary Table 4
Direct comparison of winning PANDA challenge algorithm and developed algorithm
Task: Gleason grading (agreement analysis).

**Grading dataset (pathologists n=)**	**Quadratically weighted kappa, average PANDA algorithm***	**Quadratically weighted kappa, average OWN algorithm**
**WNS (n=11)**	0.69	0.72
**UKK (n=10)**	0.72	0.77

Comment: * Using only Part 1 of the ensemble (three networks) – full ensemble (eight networks) performed substantially worse (e.g., for WNS dataset quadratically weighted kappa = 0.56).

Supplementary Table 5
Performance of the developed algorithm for tumor detection using a detection probability cut-off optimized for high sensitivity.

**Tumor detection dataset**	**Tumor probability cutoff 0.5 [ACC, F1, PPV, NPV, SENS, SPEC]**
**ACH**	[0.941, 0.918, 0.853, 0.997, 0.994, 0.915]
**BRA**	[0.858, 0.776, 0.634, 1.000, 1.000, 0.812]
**TRO**	[0.871, 0.902, 0.822, 1.000, 1.000, 0.679]
**UKK**	[0.840, 0.736, 0.585, 0.997, 0.992, 0.795]
**WNS HAMA**	[0.840, 0.765, 0.621, 0.997, 0.994, 0.786]
**WNS LEICA**	[0.917, 0.859, 0.759, 0.996, 0.988, 0.892]

Supplementary Table 6
Performance of the developed algorithm for tumor detection (case-level metrics; tumor probability cut-off 0.85)

Tumor detection dataset	Accuracy	Sensitivity
**ACH**	1.0	1.0
**BRA**	0.867	1.0
**TRO**	0.940	1.0
**UKK**	0.902	1.0
**WNS HAMA**	0.825	1.0
**WNS LEICA**	0.926	1.0

Supplementary Figures

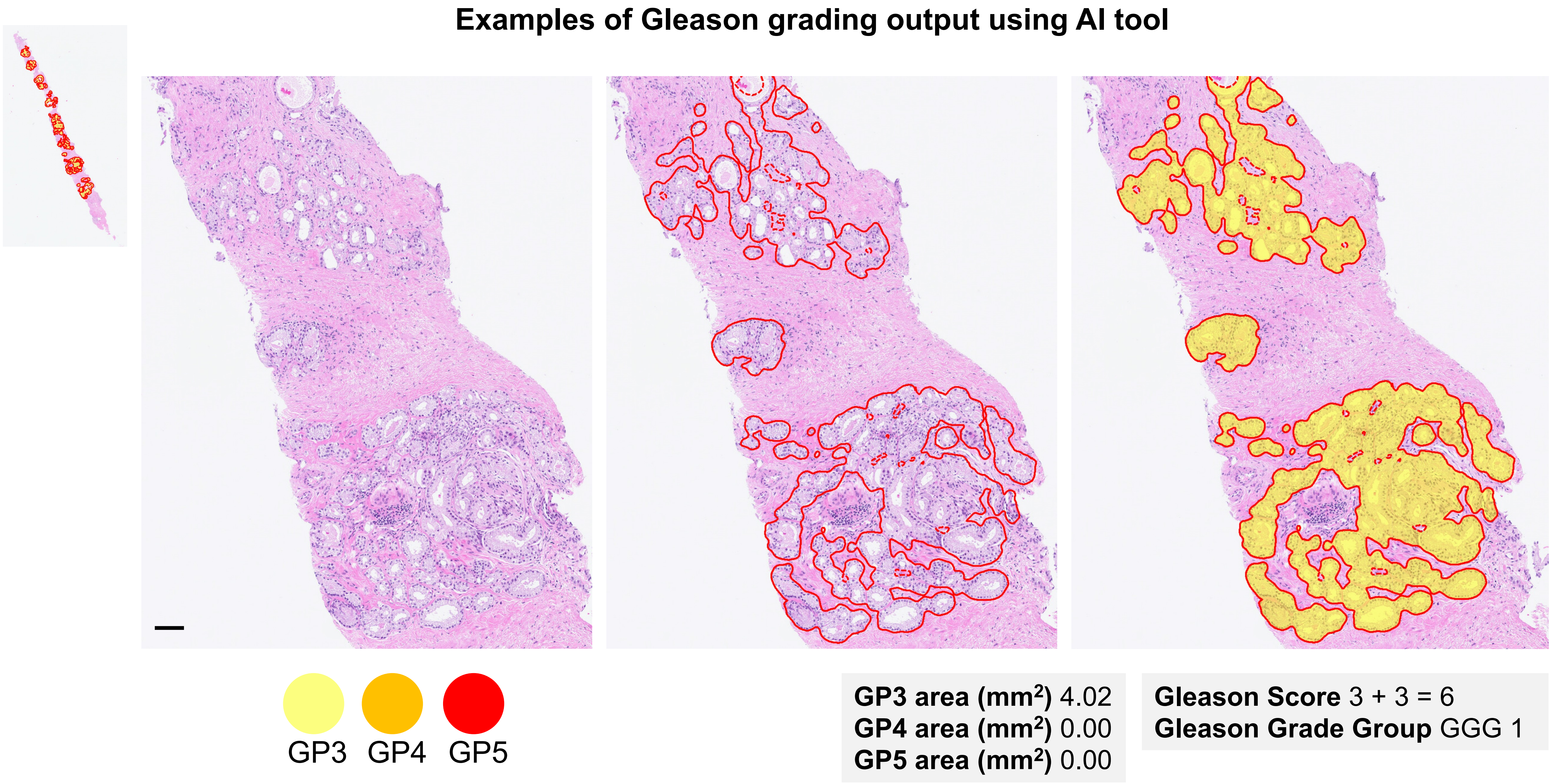

Supplementary Figure 1

Example of output of the Gleason grading module of the AI tool (1). Gleason Score 3 + 3 = 6 (Grade group 1). Scale bar for all images: 100 μm.

Examples of Gleason grading output using AI tool

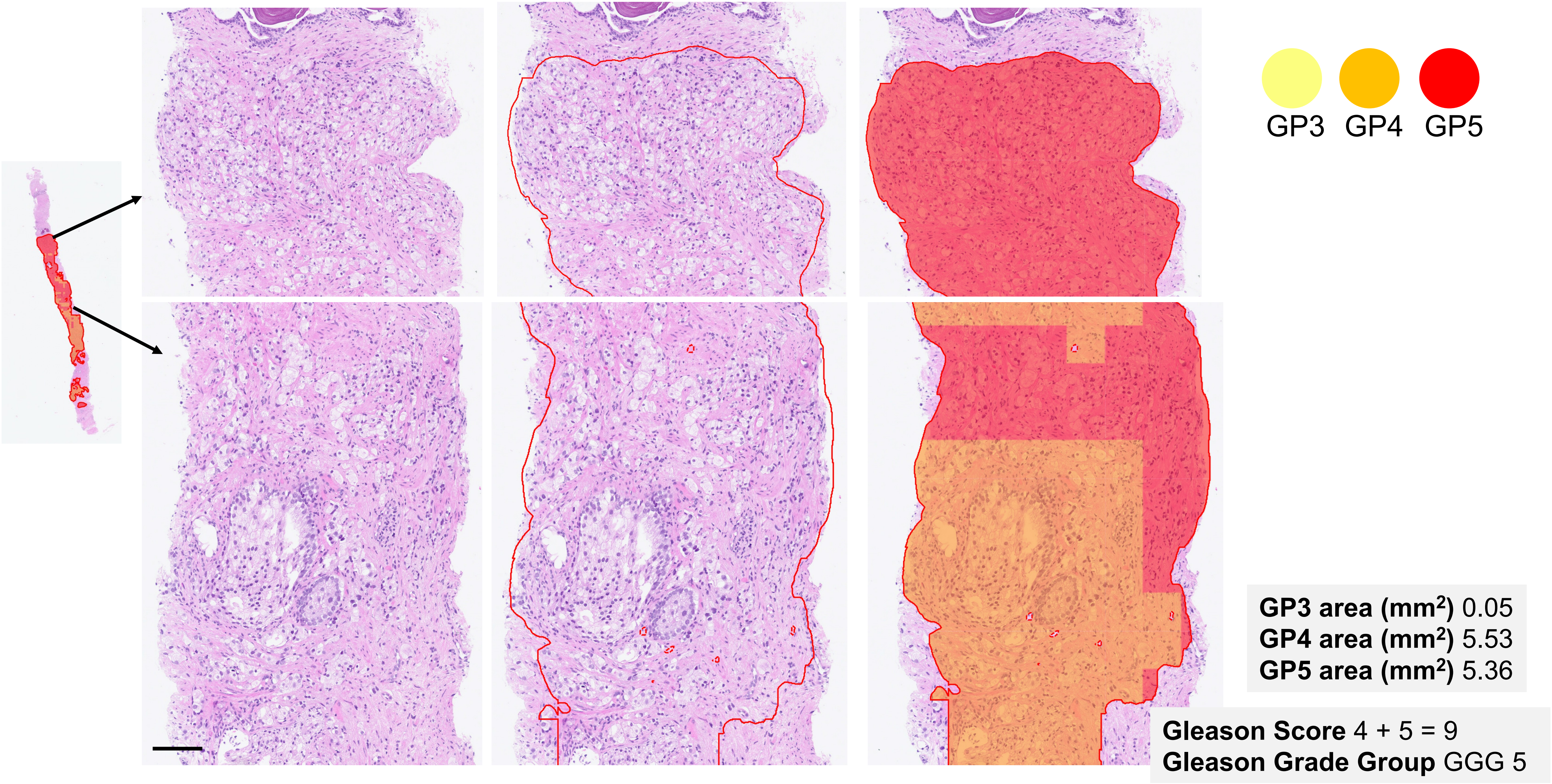

Supplementary Figure 2

Example of output of the Gleason grading module of the AI tool (2). Gleason Score 4 + 5 = 9 (Grade group 5). Scale bar for all images: 100 μm.

Suppl. Figure 3

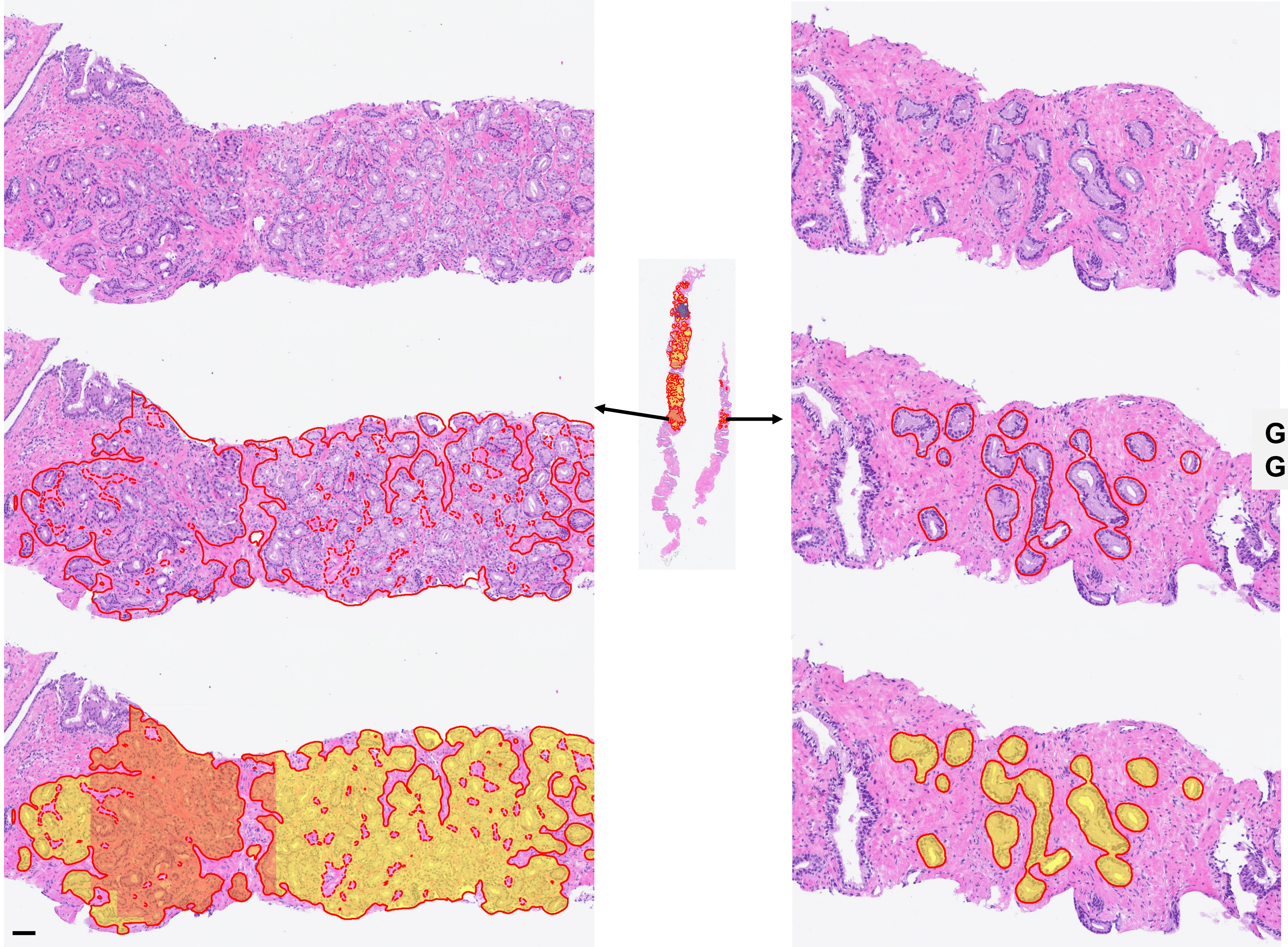

GP3 GP4 GP5

GP3 area (mm²) 2.94
GP4 area (mm²) 1.39
GP5 area (mm²) 0.00

Gleason Score 3 + 4 = 7a
Gleason Grade Group GGG 2

Supplementary Figure 3
Example of output of the Gleason grading module of the AI tool (3). Gleason Score 3 + 4 = 7a (Grade group 2). Scale bar for all images: 100 μm.

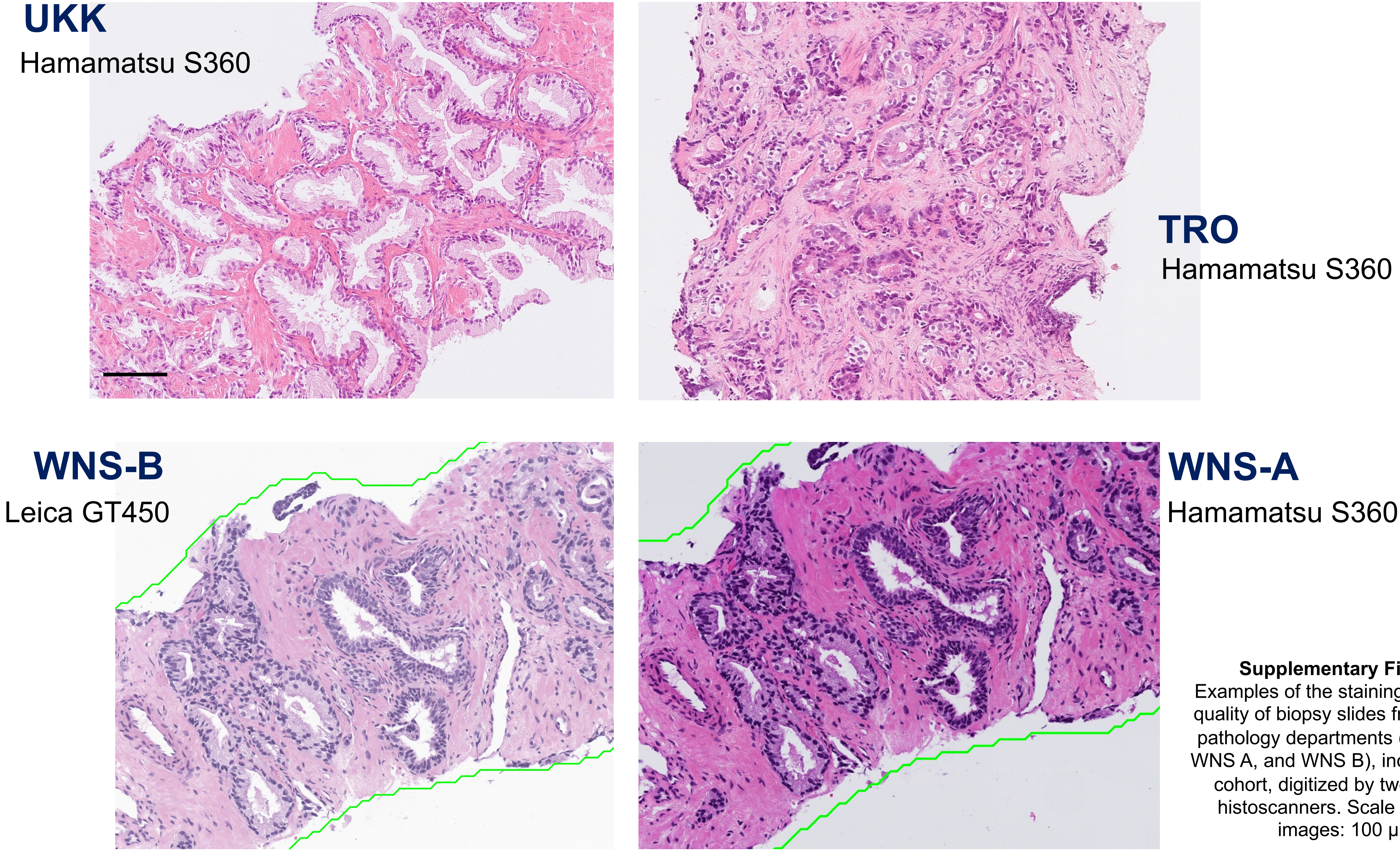

Supplementary Figure 4
Examples of the staining and cutting quality of biopsy slides from different pathology departments (UKK, TRO, WNS A, and WNS B), including WNS cohort, digitized by two different histoscanners. Scale bar for all images: 100 µm.

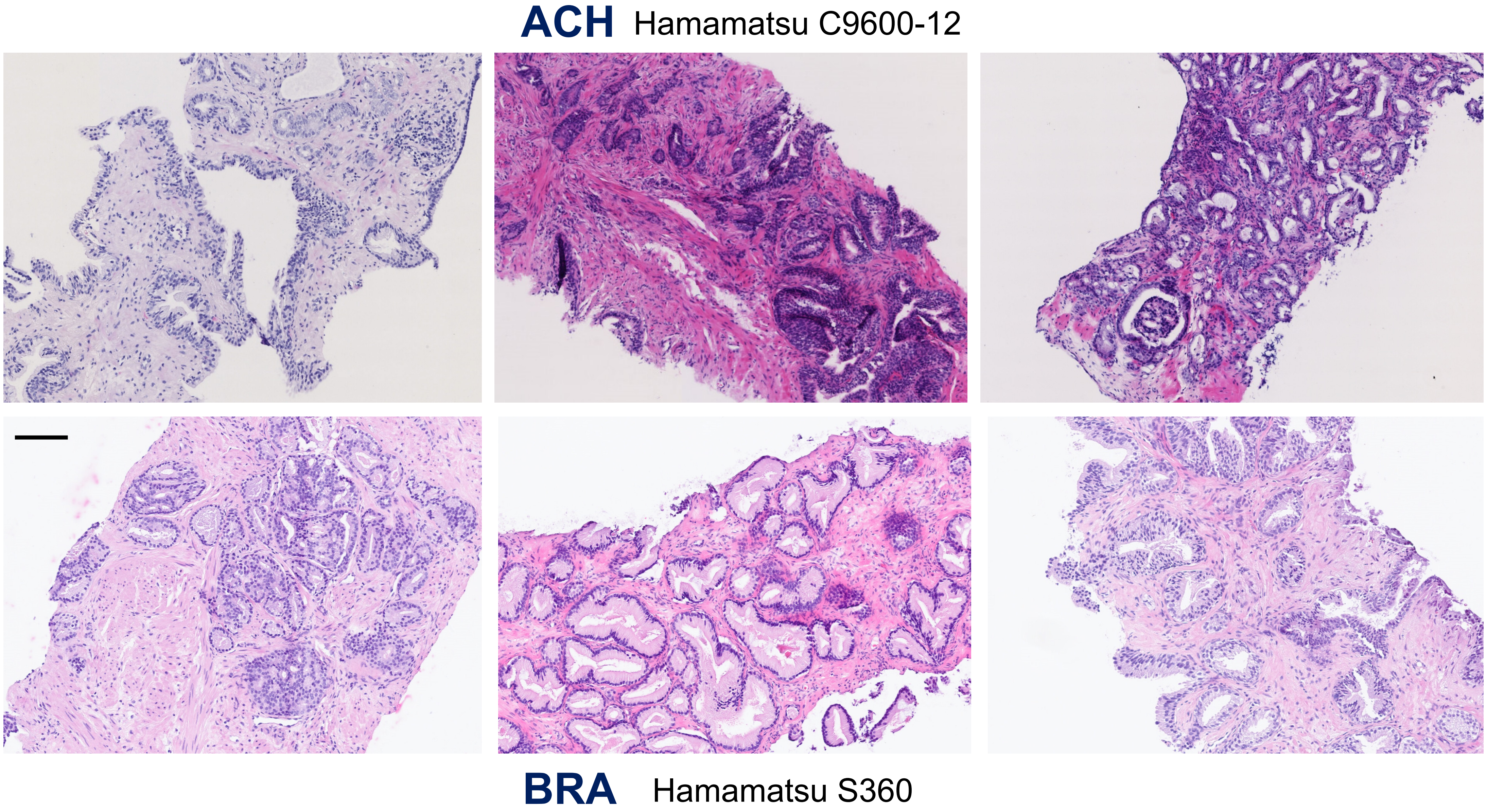

Supplementary Figure 5

Examples of the staining and cutting quality of biopsy slides from different pathology departments (ACH and BRA). Scale bar for all images: 100 µm

Additional examples of false negative tumor detections

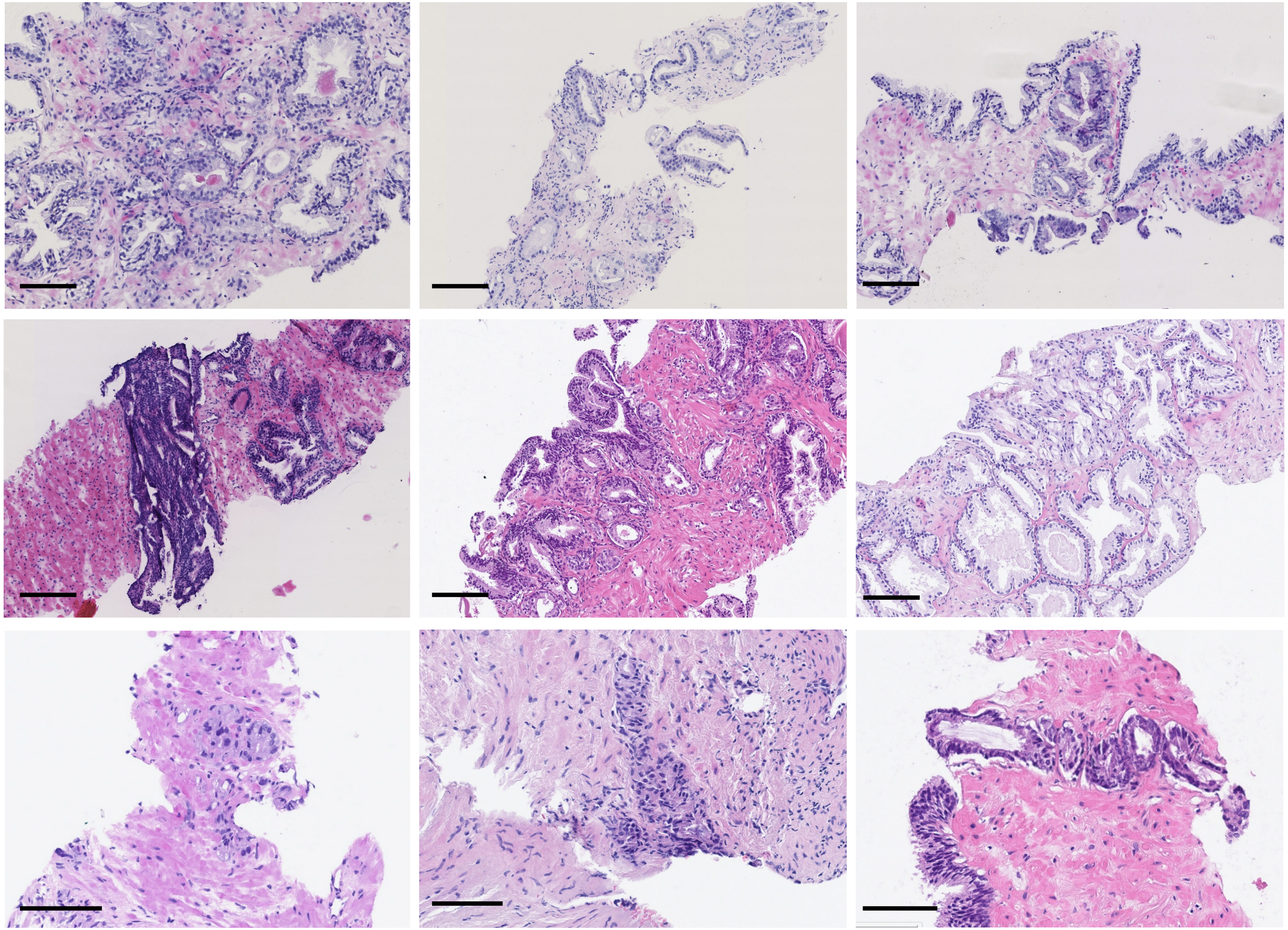

Supplementary Figure 6

Additional examples of false negative tumor detections. Scale bars: 100 μm.

Additional examples of false positive tumor detections/alerts

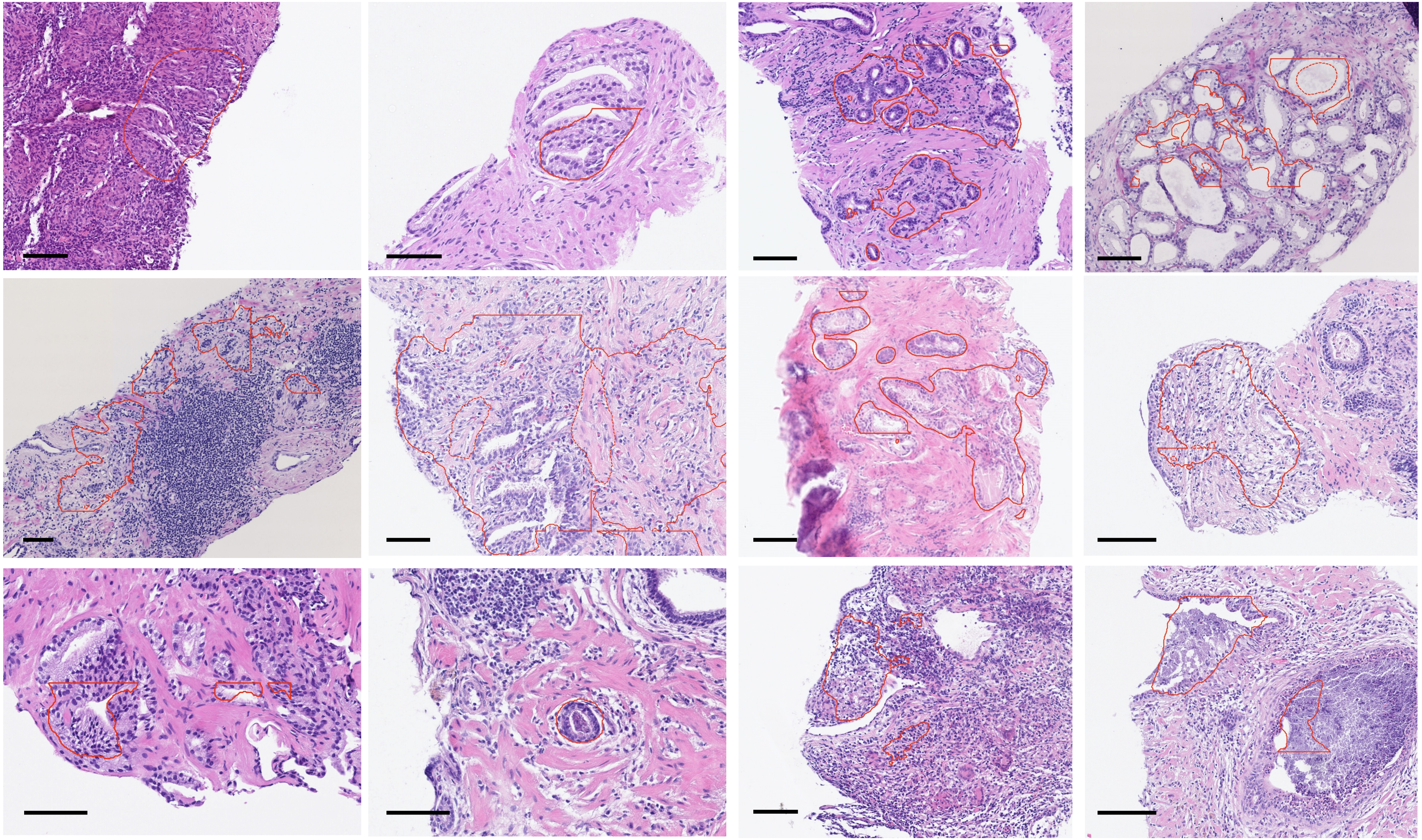

Supplementary Figure 7 Additional examples of false positive tumor detections/alerts. Scale bars: 100 μm.

Examples of tumor regions missed by pathologists, detected by AI tool (WNS)

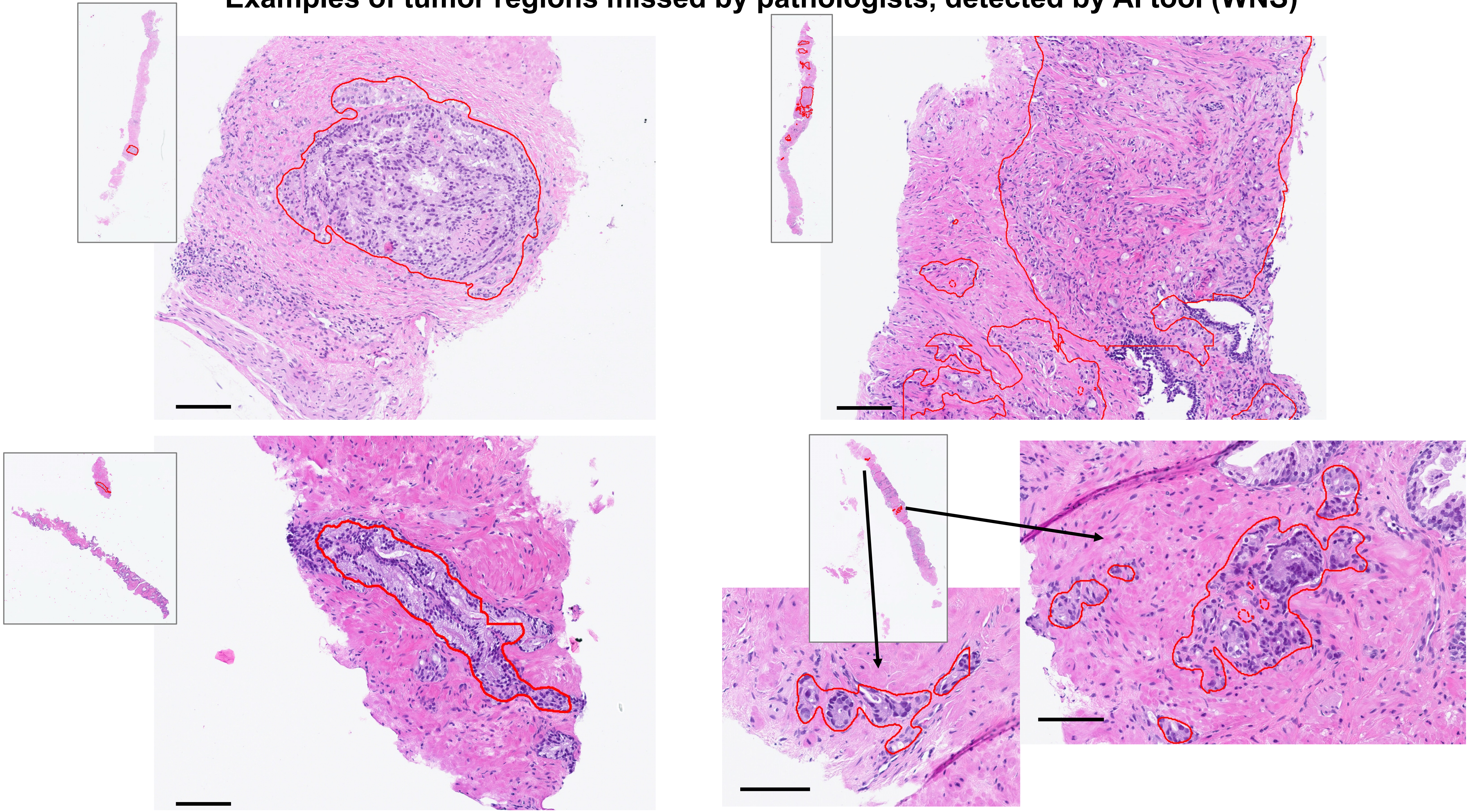

Supplementary Figure 8 Examples of tumor regions missed by pathologists, detected by AI tool (WNS cohort). Scale bars: 100 µm.

Examples of tumor regions missed by pathologists, detected by AI tool (UKK)

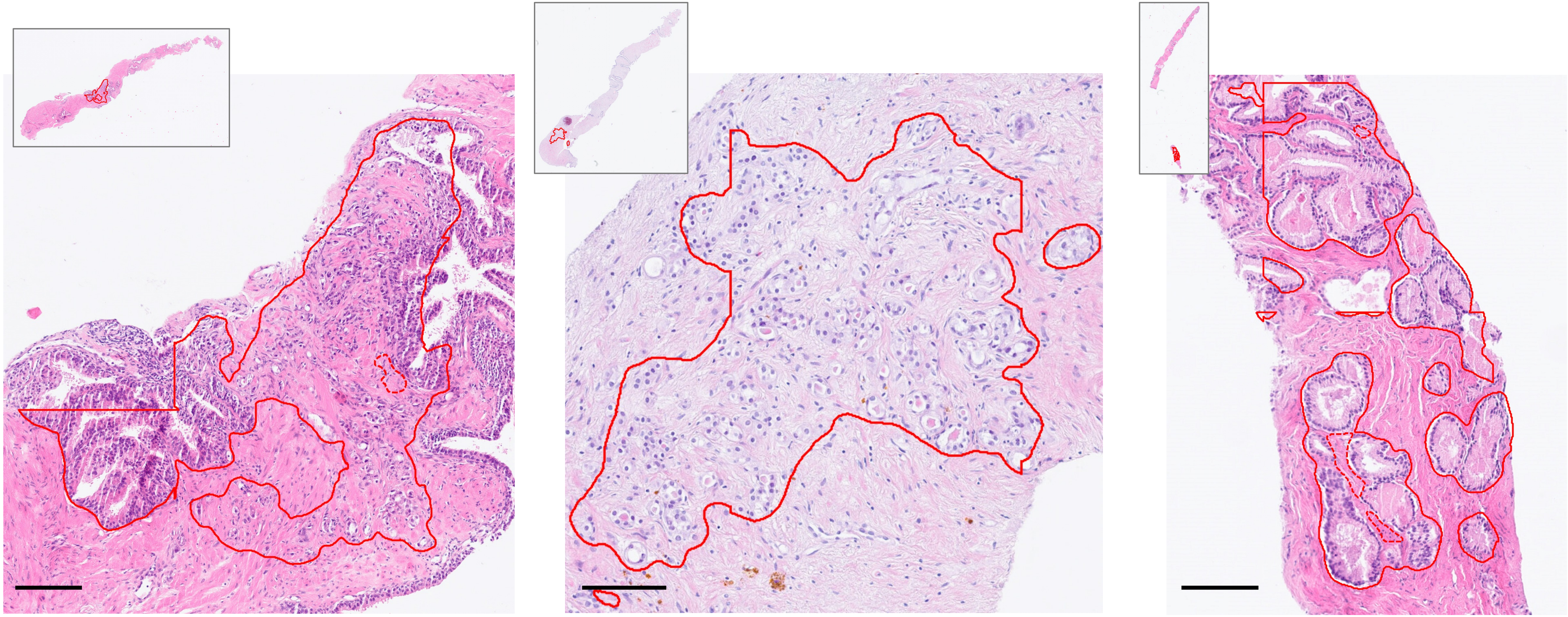

Supplementary Figure 9 Examples of tumor regions missed by pathologists, detected by AI tool (UKK cohort). Scale bars: 100 μm.

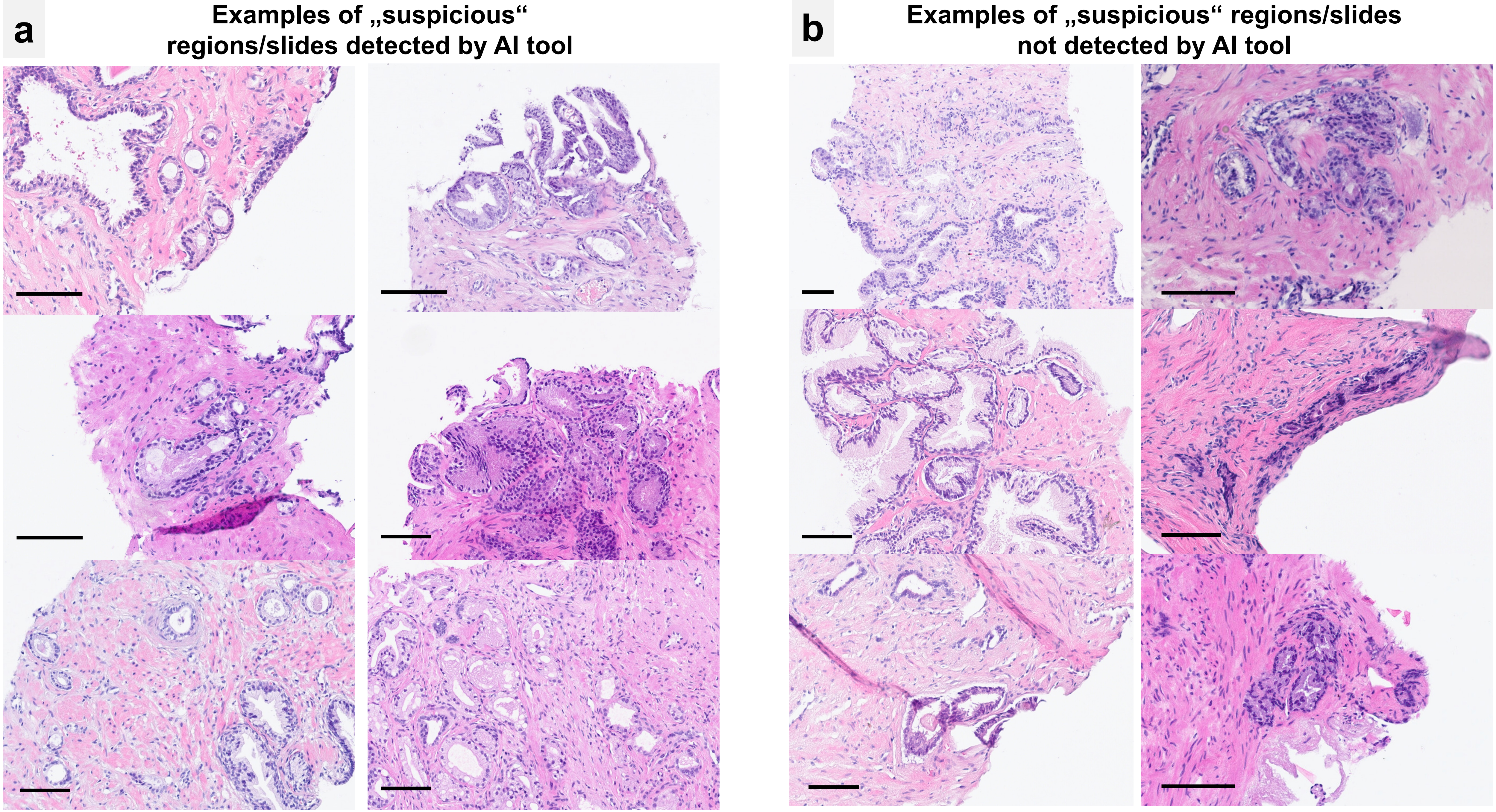

Supplementary Figure 10 Examples of regions/slides classified as „suspicious“ by pathologists during central review and detected by AI tool as having high probability of being a tumor (a) or not detected by AI tool as having high probability of being tumor (b). Scale bars: 100 µm.

a UKK (n = 227)

Votes	3	4	5	6	7	8	9	10
**Tumor area / slide, mm²**								
Mean	0.5	1.7	2.4	2.4	2.2	1.7	2.9	4.2
SD	0.3	2.8	2.4	2.8	2.5	2.1	3.5	4.4
**Tumor area / slide, mm²**								
Mean	2.1			2.5				
SD	3.0			2.5				

Votes 3-10:
Pearson's correlation 0.14 (0.01-0.27), p = 0.03.

Votes 3-5 vs. 6-10:
Wilcoxon test's p = 0.310

b WNS (n = 159)

Votes	3	4	5	6	7	8	9	10
**Tumor area / slide, mm²**								
Mean	0.3	3.4	2.6	2.3	5.9	2.4	8.4	6.2
SD	0.3	3.9	3.6	2.2	7.4	2.5	6.6	7.6
**Tumor area / slide, mm²**								
Mean	4.2			2.8				
SD	5.6			3.7				

Votes 3-10:
Pearson's correlation 0.24 (0.08-0.38), p = 0.003.

Votes 3-5 vs. 6-10:
Wilcoxon test's p = 0.016

Supplementary Figure 11 Dependence of agreement among pathologists on absolute tumor area in analyzed slides: a. UKK cohort. b. WNS cohort. A positive correlation is evident implying that higher volume of tumor in a biopsy slide leads to higher agreement among Gleason grading of single pathologists. The effect of correlation is, however, very small.

a **Confusion tables for Gleason grading (AI tool against majority of pathologists vote)**

n = 227 UKK

AI tool	Majority of votes: GG1	GG2	GG3	GG4	GG5
GG1	54	12	1	0	0
GG2	7	44	1	0	0
GG3	2	14	18	1	1
GG4	3	2	9	9	0
GG5	0	2	6	6	35

b **Confusion tables for Gleason grading (AI tool against majority of pathologists vote)**

n = 157 WNS

AI tool	Majority of votes: GG1	GG2	GG3	GG4	GG5
GG1	25	8	3	0	0
GG2	9	18	4	0	0
GG3	2	5	19	3	1
GG4	2	1	5	5	3
GG5	0	2	10	11	23

c **Quadratic kappa for AI tool against consensus grading of pathologists (GG 1 vs GG 2-5)**

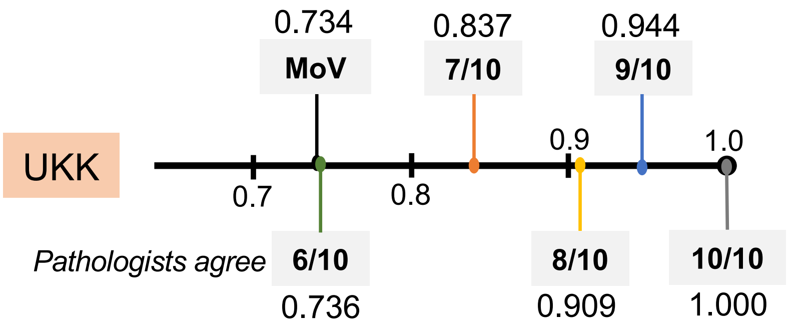

Bx	n=
MoV	227
6/10	151
7/10	107
8/10	72
9/10	38
10/10	20

d **Quadratic kappa for AI tool against consensus grading of pathologists (GG 1 vs GG 2-5)**

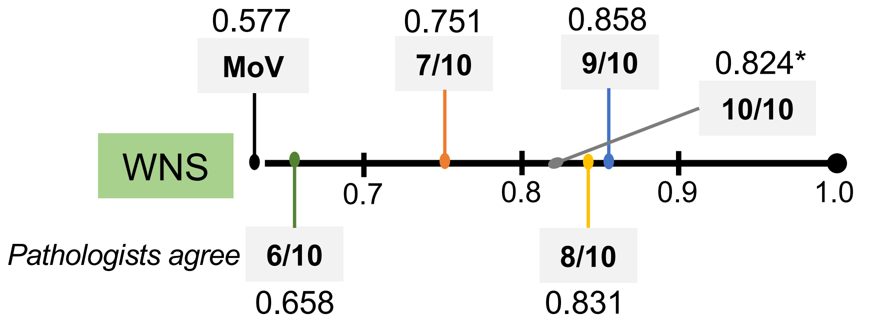

Bx	n=
MoV	159
6/10	122
7/10	89
8/10	50
9/10	34
10/10	18

*GG1 biopsies n = 3

Supplementary Figure 12 Confusion tables for Gleason grading for the whole cohorts (a, b) and agreement analysis for slides with Grade group 1 vs Grade Groups 2-5 (c, d). **a, b:** Confusion tables for Gleason grading for the whole cohorts (a: UKK, b: WNS): AI tool against majority of votes. As in UKK cohort (graded by 10 pathologists) approximately 30 cases have equivocal quantity of majority votes for two grade groups simultaneously, grading of AI tool was added to produce unambiguous distribution data. For WNS cohort (graded by 11 pathologists) majority of votes classification resulted in unambiguous classification without adding AI tool grading results. **c, d**: Agreement analysis for biopsy slides with Grade group 1 vs Grade Groups 2-5. Substantial increases in agreement of AI tool with consensus of pathologists was evident (at least 6 votes for the same Grade group), especially in cases where > 6 pathologists were agreeing on the Grade group for single cases.

Gleason grading in cases without consensus among pathologists (cohort UKK)

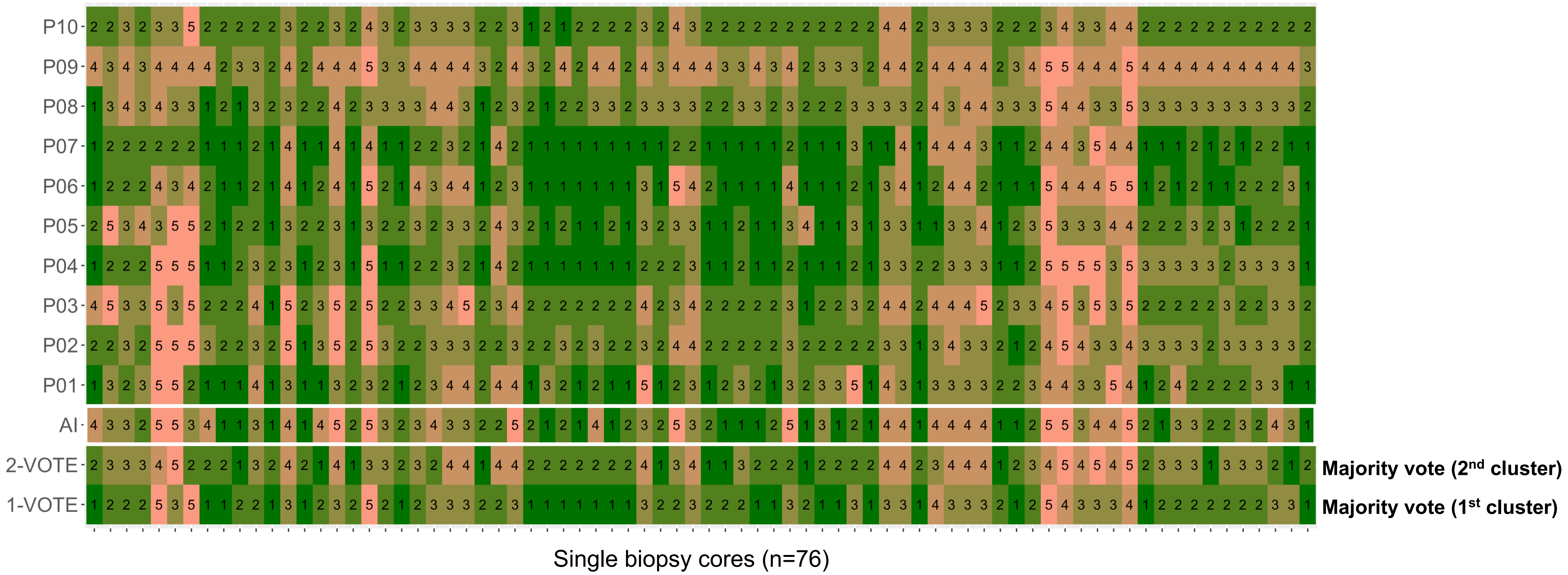

Supplementary Figure 13 Detailed Gleason grading results for AI tool and pathologists in cases without consensus (<6 pathologists provided the same Gleason grading) among pathologists (cohort UKK). Abbreviations: 1-VOTE – majority of votes, 2-VOTE – second most preferred Grade group (second cluster of pathologists' votes). In 30 cases of UKK cohort number of pathologists' votes for first and second cluster was the same.

Gleason grading in cases without consensus among pathologists (cohort WNS)

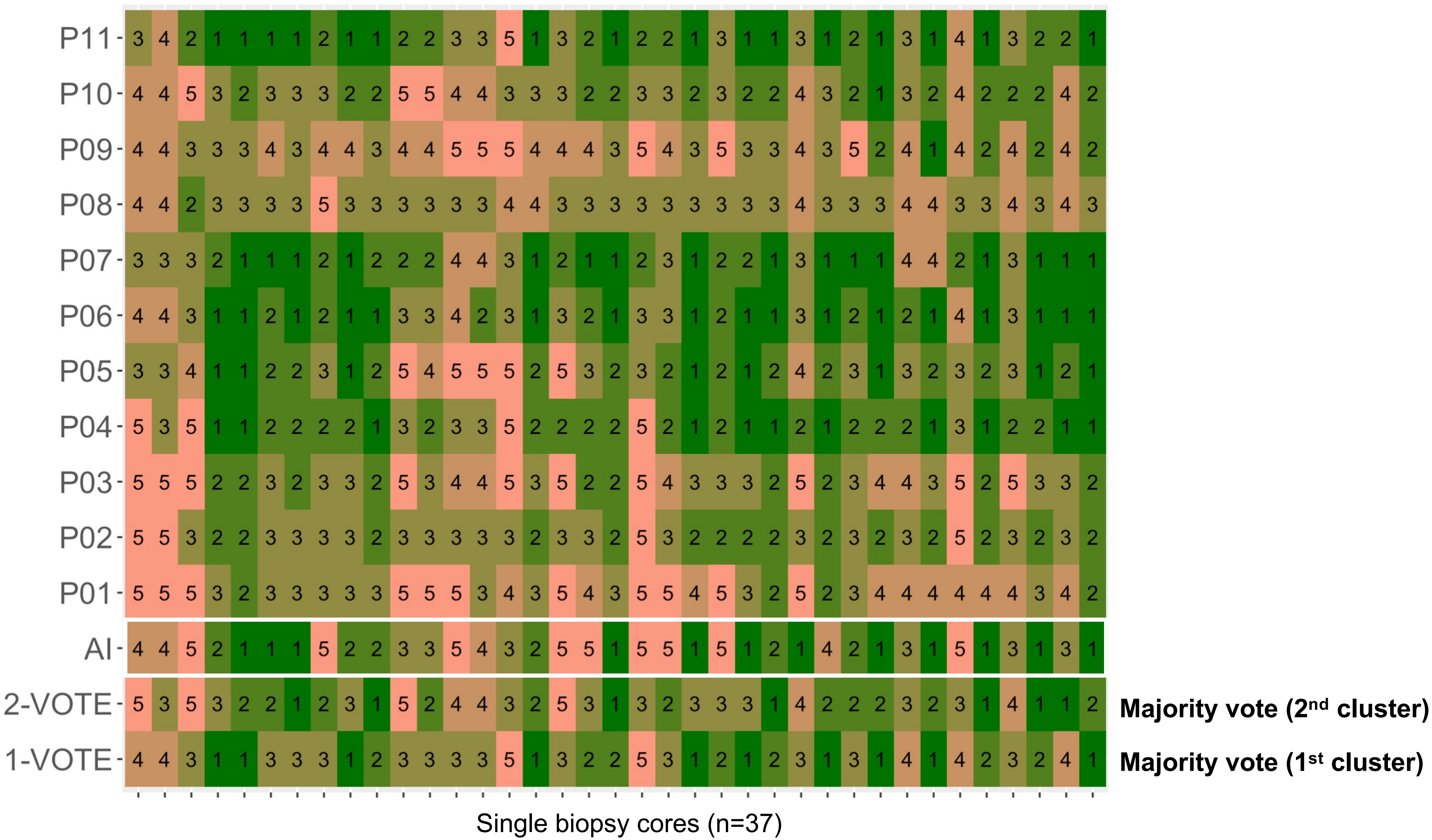

Supplementary Figure 14 Detailed Gleason grading results for AI tool and pathologists in cases without consensus (<6 pathologists provided the same Gleason grading) among pathologists (cohort WNS). Abbreviations: 1-VOTE – majority of votes, 2-VOTE – second most preferred Grade group (second cluster of pathologists' votes).

a **UKK cohort**

	Quadratic Kappa value		
	**Pathologist**	**AI tool**	**Cores n=**
**P1**	0.834	0.935	143 / 227
**P2**	0.927	0.910	142 / 227
**P3**	0.902	0.903	140 / 227
**P4**	0.938	0.921	140 / 227
**P5**	0.931	0.921	136 / 227
**P6**	0.951	0.914	138 / 227
**P7**	0.881	0.906	150 / 227
**P8**	0.849	0.932	145 / 227
**P9**	0.744	0.896	157 / 227
**P10**	0.893	0.896	157 / 227

Calculation of majority of votes based on grading of 9 pathologists (all cores with 5 votes for single Gleason score)

b **WNS cohort**

	Quadratic Kappa value		
	**Pathologist**	**AI tool**	**Cores n=**
**P1**	0.802	0.868	80 / 159
**P2**	0.914	0.879	71 / 159
**P3**	0.878	0.894	75 / 159
**P4**	0.880	0.903	76 / 159
**P5**	0.899	0.896	69 / 159
**P6**	0.849	0.906	76 / 159
**P7**	0.873	0.891	73 / 159
**P8**	0.827	0.886	81 / 159
**P9**	0.792	0.862	83 / 159
**P10**	0.913	0.877	74 / 159
**P11**	0.944	0.877	74 / 159

Calculation of majority of votes based on grading of 10 pathologists (all cores with 6 votes for single Gleason score)

Supplementary Figure 15 The results of Gleason grading with quadratically weighted kappa values between single pathologist and a group of pathologists excluding this single pathologist (based on cases where > 50% of pathologists provided the same Gleason score). The kappa value for AI tool against consensus of the same group of pathologists excluding one is shown in parallel.

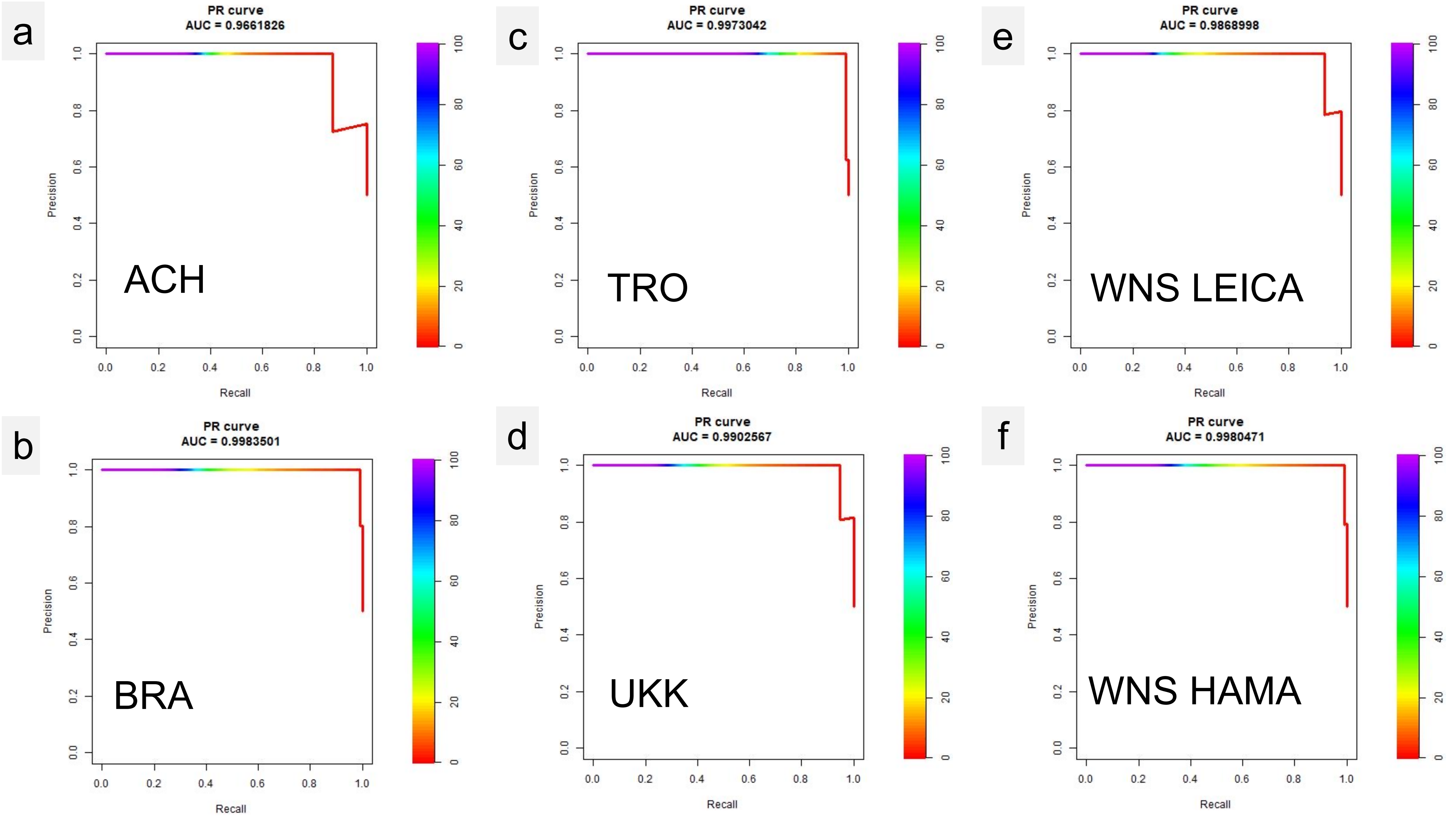

Supplementary Figure 16 Area under precision-recall curve analysis for different tumor probability thresholds illustrating model precision and recall at different probability cut-offs, for different study cohorts: a) ACH, b) BRA, c) TRO, d) UKK, e) WNS LEICA, f) WNS HAMA.

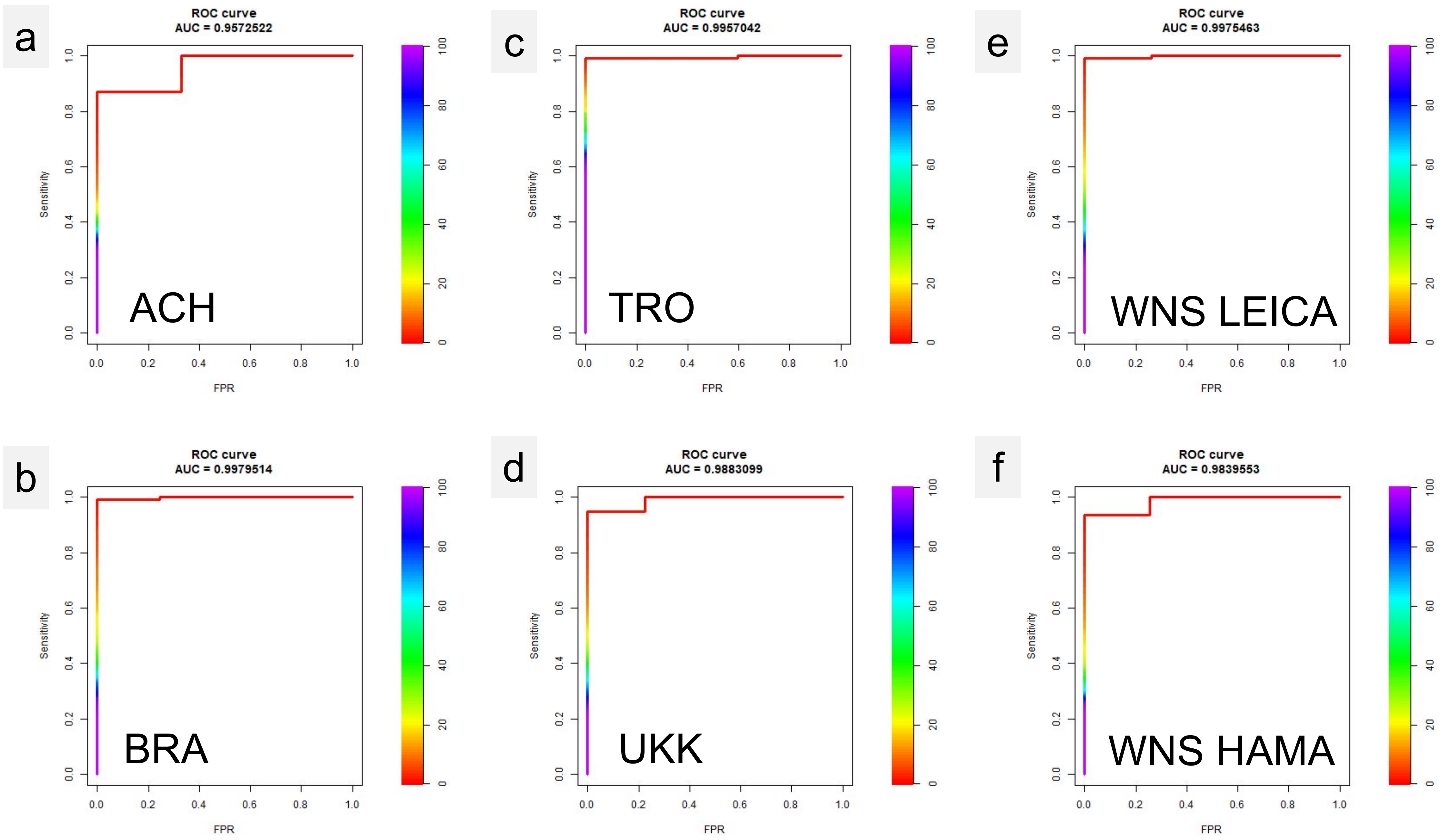

Supplementary Figure 17 Area under receiver operating curve analysis for different tumor probability thresholds illustrating model sensitivity and specificity at different probability cut-offs, for different study cohorts: a) ACH, b) BRA, c) TRO, d) UKK, e) WNS LEICA, f) WNS HAMA.
